# The role of gravitational body forces in the development of metamorphic core complexes

**DOI:** 10.1038/s41467-022-33361-2

**Published:** 2022-09-26

**Authors:** Alireza Bahadori, William E. Holt, Jacqueline Austermann, Lajhon Campbell, E. Troy Rasbury, Daniel M. Davis, Christopher M. Calvelage, Lucy M. Flesch

**Affiliations:** 1 Lamont-Doherty Earth Observatory, Columbia University in the City of New York, Palisades, NY USA; 2 Department of Geosciences, Stony Brook University, Stony Brook, NY USA; 3 Department of Earth and Atmospheric Sciences, University of Houston, Houston, TX USA; 4 Department of Earth, Atmospheric, and Planetary Sciences, Purdue University, West Lafayette, IN USA

**Keywords:** Tectonics, Geodynamics

## Abstract

Within extreme continental extension areas, ductile middle crust is exhumed at the surface as metamorphic core complexes. Sophisticated quantitative models of extreme extension predicted upward transport of ductile middle-lower crust through time. Here we develop a general model for metamorphic core complexes formation and demonstrate that they result from the collapse of a mountain belt supported by a thickened crustal root. We show that gravitational body forces generated by topography and crustal root cause an upward flow pattern of the ductile lower-middle crust, facilitated by a detachment surface evolving into low-angle normal fault. This detachment surface acquires large amounts of finite strain, consistent with thick mylonite zones found in metamorphic core complexes. Isostatic rebound exposes the detachment in a domed upwarp, while the final Moho discontinuity across the extended region relaxes to a flat geometry. This work suggests that belts of metamorphic core complexes are a fossil signature of collapsed highlands.

## Abstract (web summary)

A long-standing controversy surrounds low-angle nature of observed detachment faults within metamorphic core complexes. Here, the authors show that post-orogenic collapse of mountain belts can create a low-angle detachment, resolving the controversy.

## Introduction

Continental or metamorphic core complexes (MCCs)^1^ as well as oceanic core complexes^1^ are found in both extensional continental settings^2–6^ and oceanic spreading centers^7–9^, respectively. Metamorphic rocks and migmatites constitute the dome core within a MCC^10,11^ that is exhumed at the surface^1,12–14^. A low-angle detachment fault (<30°) constitutes the uppermost part of the domal or arched structure of a MCC^1,12–14^. Above the detachment surface lies brittle faulted cover rock^11^, which is in direct contact with ductile middle-lower crust, with adjacent sedimentary basins formed in association with this fault geometry^1,12–14^. While the Anderson fault theory^15^ suggests that normal faults should form at high angles (>45°) under extension, the origin and mechanics of the low-angle detachment zone that forms a MCC, whether it originates with a high or low dip angle, have been highly debated^6,10,15–20^. Previous numerical simulations of MCC formation and evolution^13,21–30^ have tried to explore the mechanisms and conditions of MCC formation. These models show that factors that can produce a low-angle detachment fault through an extensional boundary condition include (1) a viscosity or density contrast between the brittle upper crust and ductile middle–lower crust^26^, (2) localization of strain at a single pre-defined weak fault zone^1,10,11,23,26,31,32^ or a single fault that forms spontaneously as the result of shear localization and strain-induced weakening^13,21,25,26,33,34^, (3) partial melting associated with an increase in temperature^23,24,26,28^, or (4) a thickened crust^26,31^.

**Fig. 1:**
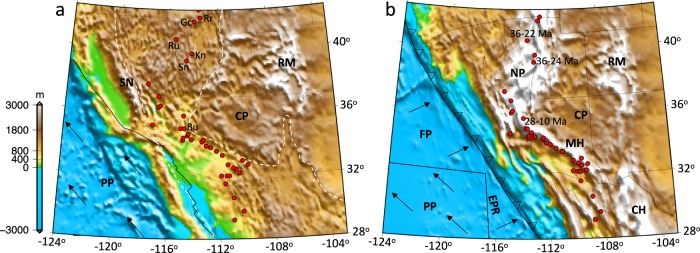
The high gravitational potential energy of a mountain chain in southwestern North America generated deviatoric tensional stresses for extensional collapse and formation of metamorphic core complexes. **a** The present-day surface elevation within the Basin and Range Province of southwestern North America (area surrounded by dashed line) together with present-day locality of metamorphic core complexes, shown with red dots. Gc Grouse Creek, Rr Raft River, Ru Ruby, Kn Kern, Sn Snake Range, Bu Buckskin. **b** The paleo-topography model at the late Eocene from ref. 40, showing a continuous highland chain, with an average elevation of ~4 km, between northern Nevada to southeast Arizona and northern Mexico. The thickened crustal welt that partly supported this mountain chain was likely a consequence of Sevier-Laramide convergence. Note that the proximity of restored locations of metamorphic core complexes along the chain of high paleotopography suggests a causal link between high topography, crustal root, and metamorphic core complex occurrence in southwestern North America. Numbers represent timing of tectonic denudation for the Snake Range, Ruby, and Buckskin metamorphic core complexes from Dickinson^41^. NP Nevadaplano, RM Rocky Mountains, MH Mogollon Highlands, CH Chihuahua Highlands, CP Colorado Plateau, SN Sierra Nevada, PP Pacific Plate, FP Farallon Plate, EPR East Pacific Rise. The map images were created by authors using: www.soest.hawaii.edu/gmt/.

The complex tectonic history, dramatic extension, and large number of detailed field observations and geophysical data^4,35^ make the Basin and Range Province (BRP) of southwestern North America (SWNA) an ideal natural laboratory for studying MCC formation^4,11,36^. Within the SWNA, reconstruction of crustal structure^37^ based on palinspastically restored extension and shear history estimates over time^38^ suggests that the distribution of MCCs lie within zones or belts where the crust was thickened prior to extensional collapse (Fig. 1). Recent numerical modeling efforts^39,40^ have shown that the crustal extension and topographic collapse in SWNA was caused by tensional deviatoric stresses associated with high gravitational potential energy (GPE) of this mountain chain, as plate motion boundary conditions transitioned from subduction to transform motion and as the lithosphere was progressively weakened by heat, fluids, and volcanism during the slab rollback history^39,40^. The proximity of restored locations of MCCs along the chain of high paleo-topography^37^ (Fig. 1) suggests a causal link between high topography, crustal roots, and MCC occurrence.

Following the periods of crustal shortening, during the Sevier-Laramide phases (~155–60 Ma)^41,42^, and shallow- to flat-subduction of the east dipping Farallon slab along the western margin of the North American plate (~70–50 Ma)^43^, the dramatic lithospheric extension of SWNA resulted in the present-day BRP (Fig. 1a). This lithospheric extension was initiated following the rollback of the Farallon Slab beginning at ~50 Ma and also following the displacement of the East Pacific Rise (EPR) beneath the southwestern margin of the North American Plate beginning at ~30 Ma^44,45^. Through this extreme extension and crustal thinning process, deep crustal rocks were exposed at the surface in MCCs^4,36^ of the Cordillera of North America (NA) (Fig. 1). In the northern section (southern Nevada and Utah to central-northern Canada), MCCs formed during ~55–20 Ma^46^, and in the central-southern section (northern Arizona to central-northern Mexico), MCCs developed during ~35–10 Ma^46^ (Fig. 1).

Within continental lithosphere, extension typically post-dates a period of horizontal crustal shortening and mountain building, accommodated within a thrust belt of finite width^41,47–52^. Such a crustal welt results in a compensating root and high GPE relative to the adjacent areas without a crustal root. To date, no time-dependent extensional thermomechanical model has accounted for the depth-dependent body force effects generated by a laterally varying surface and Moho topography. Furthermore, it is unknown how this setting of a thickened crust prior to extension can explain the evolution history of the heavily mylonitized detachment fault surface, which is typically exposed at low angles in the field. While some previous modeling studies showed that MCCs can form without a thickened crust^13,28^, in this paper we show that the formation of a MCC is attributed to post-orogenic collapse of a thickened crust by investigating the kinematics, timing, and mechanism responsible for formation of the MCCs found in the Cordillera of NA^41^ (e.g., the Ruby^53^ and Snake Range^54^ MCCs). While in the central-southern section of the Cordillera of NA the Pacific-North America motion generated a trans-tensional setting and lithospheric extension following collision of the EPR with the trench at ~30 Ma^41^, in the northern section of the Cordillera of NA, the lithospheric extension and formation of MCCs coincided with the timing of an active convergent plate boundary zone (subduction)^41^ (Fig. 1b).

Here we show that strain localization which is essential to MCCs arises when there is a period of crustal thickening prior to extension. We show that MCCs form under two conditions: (1) the free boundary collapse of a thickened crust (no prior constraint for extension) under the influences of high GPE and in the presence of basal tractions associated with the mantle convection and (2) topographic collapse of areas with high GPE and localized crustal thickening under the influences of far-field extensional stresses with basal pressure equilibrium. Both cases produce localized thinning of the upper crust, which is isostatically compensated by the flow of low-viscosity ductile lower crust into the MCC. Isostatic root rebound concentrates strain rates into a detachment that allows upward transfer of ductile middle crust. Our results show that in the absence of factors discussed by previous studies, like a pre-existing weak fault, density or viscosity anomaly, and partial melting within the crust, extensional models without a crustal root and a nearly uniform distribution of GPE produce broad distributed zones of high-angle conjugate block faulting and symmetric domes, leading to horst (range) and graben (basin) structures. However, those with a crustal root and a non-uniform distribution of GPE produce shearing along a major detachment to expose middle crust at the surface as a MCC, followed in time by the development of a horst (range) and graben (basin) structural setting.

## Results

### Numerical experiments

To investigate the role of slab rollback and a change in the thermal state of the lithosphere together with the role of gravitational body forces associated with the presence of the paleo-highlands on MCC formation in SWNA, we use time-dependent 2.5-D thermomechanical numerical experiments to investigate the main factors controlling the MCC formation in the Cordillera of NA (“Methods”). While the thermomechanical model is 2-D, by extrapolating the velocity field at the surface in the third dimension we produce a 2.5-D model that possesses a surface upon which erosion and deposition can occur (“Methods”). Taking into account the lithosphere’s dynamic influences, we evaluate quantitatively the kinematics, timing, and formation mechanism of MCCs based on estimates of gravitational body forces, temperature perturbation effect within the lithosphere following slab rollback, time-dependent traction field associated with mantle convection underneath the lithosphere, and the effects of erosion, transport, and deposition of materials at the surface (“Methods”).

We have run six models (Table 1) with varying crustal structures that include: (1) reconstructed crustal structure at the late Eocene from the model of ref. 37, (2) reconstructed surface elevation at the late Eocene from the model of ref. 37, but with a flat Moho topography at 45 km depth, and (3) a uniform thickness crust with a surface elevation of 4 km and a Moho depth of 45 km. For all models, we use the time-dependent reconstructed temperature model of ref. 40 at the base (Supplementary Fig. 1), and all models include partial melting of the deep crust (“Methods”).

**Table 1:** Description of the numerical experiments, modes of deformation, and produced tectonic regimes (fault angle was measured at a depth of 0–10 km on those faults where strain localization occurred)

Experiment	Model no.	Elevation type	Simulation time	Fault angle (°)	Mode	Tectonic regime produced
Mantle traction at the base, free slip on the sides, and partial melting	1	Crustal root support	36 Myr	8–18	MCC (36–23 Ma)	BRP: first collapse phase
	2	Dynamic support (no crustal root)	36 Myr	39–42	Symmetric conjugate fault	BRP: second collapse phase
	3	Uniform thickness crust	36 Myr	–	Rigid	CP: cratonic
Extension on the left (2.0 mm yr^−1^), basal pressure equilibrium, and partial melting	4	Crustal root support	30 Myr	11–20	MCC (30–5 Ma)	BRP: first collapse phase
	5	Dynamic support (no crustal root)	30 Myr	30–46	Dome-like structure	NC: extensional detachment faulting
	6	Uniform thickness crust	30 Myr	43–44	Pure shear	BRP: second collapse phase

*BRP* Basin and Range Province, *CP* Colorado Plateau, *MCC* metamorphic core complex, *NC* Northern Cordillera.

**Fig. 2:**
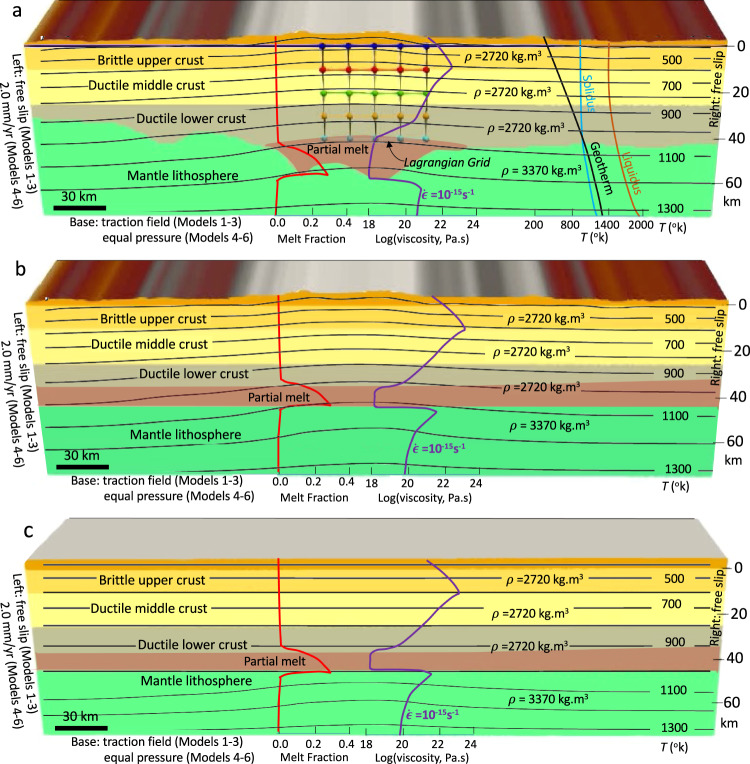
Model set-up and boundary conditions for the thermomechanical simulations that include partial melting in the deep crust. **a** Model geometry, parameters and boundary conditions, temperature, and strength as a function of depth for the experiments with a crustal root together with the locations of Lagrangian markers used to track deformation history and finite strain in the central part of the model (E–W cross-section is at 38° N from the model of ref. 37). **b** Model geometry, parameters and boundary conditions, temperature, and strength as a function of depth for the experiments with a flat Moho topography and a laterally varying surface elevation. **c** Model geometry, parameters and boundary conditions, temperature, and strength as a function of depth for the experiments with a uniform thickness crust (a flat Moho topography and a flat surface elevation); All models are 75 km deep. The purple graph represents the defined starting viscosities within the lithosphere based on a constant strain rate of 1 e^−15^ s^−1^. The red graph represents the magnitude of partial melting within the crust. The black, blue, and orange graphs represent the geotherm, solidus, and liquidus, respectively.

To investigate the effect that deeper mantle dynamics have on lithosphere deformation and MCC formation in SWNA, for the first three simulations (models 1–3), we use a free slip boundary condition on the left and right sides of the model together with a stress boundary condition at the base associated with mantle convection since the late Eocene (Fig. 2) (“Methods”). We investigate the evolution of mantle flow below the North American lithosphere based on backward simulation of the global mantle convection to the late Eocene using the global density distribution model TX2008^55,56^ (Supplementary Fig. 2), representing present-day mantle and lithospheric density variations (Methods). We determine estimates of spatial and temporal variations in deviatoric stress tensors and the associated tractive field at different depths from the time-dependent mantle convection model. For the thermomechanical model of the lithosphere, we employ the time-dependent sub-lithosphere tractions (stresses) (Supplementary Fig. 3) as the basal stress boundary conditions. The lithosphere model then changes the flow across the border of our finite element mesh to maintain the specified stress field at the base over time, which is connected to the deeper mantle dynamics.

To investigate the far-field extensional stress effects on lithosphere deformation and MCC formation, for the second three simulations (models 4–6), we use a constant velocity boundary condition of 2 mm yr^−1^ on the left side of the finite element mesh, a free slip boundary condition on the right side, and we apply an equal pressure boundary condition at the base (Fig. 2) (“Methods”). We couple all the thermomechanical models with a surface processes model (“Methods”) to provide a realistic mass redistribution from erosion, transport, and deposition to be included in the evolving thermomechanical model.

### Body force and mantle flow influences on lithosphere deformation and MCC formation (models 1–3)

Our simulations, under the influences of different initial distributions of gravitational body forces, along with the time-dependent basal stress boundary conditions associated with mantle flow effects, show three end-member deformation modes that include: (1) the MCC mode, demonstrated by the formation of a low-angle detachment fault and exhumation of the ductile middle crust; this mode evolves eventually to horst and graben topography; (2) the symmetric conjugate fault mode, demonstrated by the formation of high-angle conjugate faults, that leads to a horst and graben topography; (3) the rigid (no-deformation) mode, demonstrated by minor deformation to the crust without formation of normal faults, which leads to a flat topography. Below we describe the main characteristics of these modes.

#### The MCC mode (model 1)

**Fig. 3:**
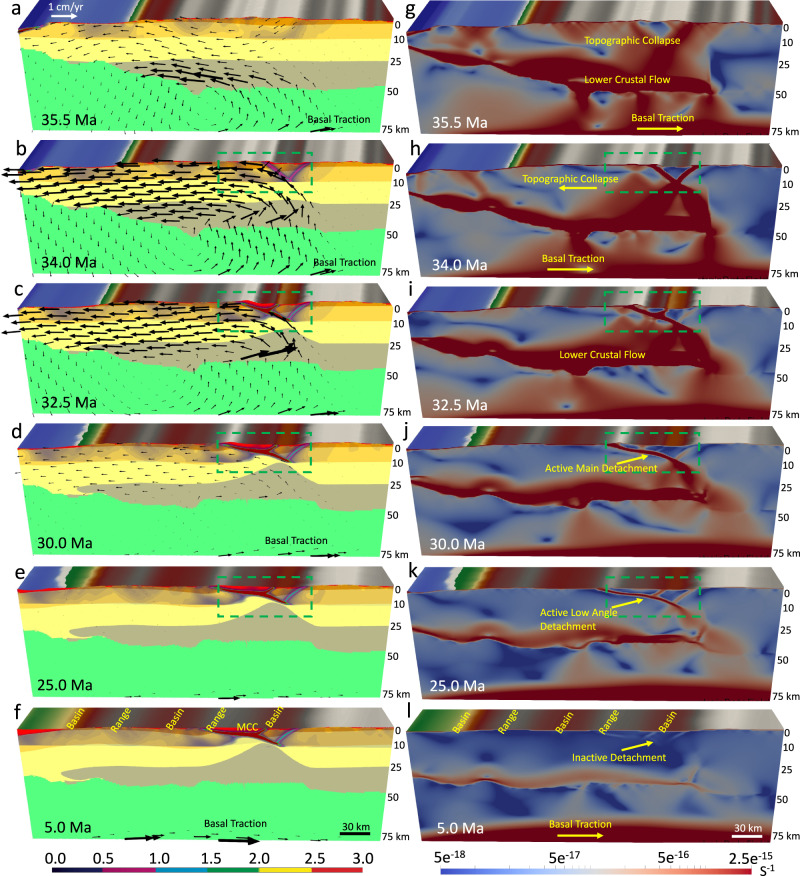
Deformation of the model with a crustal root and a laterally varying surface elevation since the late Eocene under the influence of basal tractions, free slip side boundaries, and non-uniform distribution of gravitational body forces. **a**–**f** The evolution of crustal structure together with the magnitude of the finite plastic strain for the brittle upper crust at 35.5, 34, 32.5, 30, 25, and 5 Ma, respectively. Vectors represent the flow field through time. **g**–**l** The evolution of finite strain (values represent second invariant of strain tensor) magnitude through the brittle and ductile zones of the crust at 35.5, 34, 32.5, 30, 25, and 5 Ma, respectively. Cross-section (starting geometry) is at 38° N from ref. 37. Note that in experiment with a crustal root the weakening and damage of the brittle upper crust and the resulting accumulated plastic strain and plastic shear zones produce basins and ranges. A necking center below the highlands forms and remains active. Dip of the active shear zones is high at the onset of topographic collapse and evolves into low-angle detachment fault through time. Red areas on the left panels represent locations affected by erosion and sedimentation. Experiments start at 36 Ma and evolve to 0 Ma. Stages of evolution are given in Ma. Green dashed line represents the zoomed-in area for panels in Fig. 4.

**Fig. 4:**
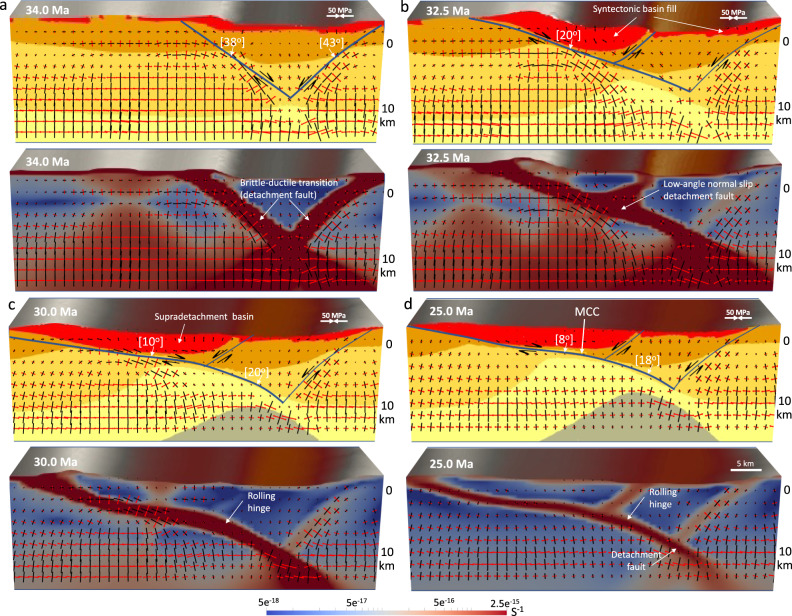
Within zones of high gravitational body forces, the relaxation of the crustal root generates a “rolling hinge” mode within the middle and lower crust. **a**–**d** Zoom-in of boxed region in Fig. 3, illustrating strain concentration and the rotation of principal axes of deviatoric stresses within the upper crust through high- and low-angle normal faulting under the influence of basal tractions, free slip side boundaries, and non-uniform distribution of gravitational body forces. Red vectors represent tensional and black vectors represent compressional principal axes of deviatoric stresses. Black arrows show sense of slip offset accumulation on the detachment. Finite strain (values represent second invariant of strain tensor) develops in response to extension and fault rotation. The blue lines denote where main and conjugate detachments, respectively, remain active. Note that in the experiment with a crustal root, a reduction in crustal thickness in response to isostatic rebound of the crustal root and collapse of topography increases the local strains within a necking zone which accommodates the doming and exhumation of the middle crust at the surface, with the numbers showing their dip angles in degrees. Note the decrease of dip angle from 38° to 20° to 8° of the asymmetric shear zone, showing the rolling hinge mode for metamorphic core complex formation. Red areas on the top panels represent locations affected by both erosion and sediment accumulation. Experiments start at 36 Ma and evolve to 0 Ma. Stages of evolution are given in Ma. MCC metamorphic core complex.

The model with a crustal root and non-uniform distribution of GPE at the late Eocene demonstrates the formation of a MCC. The imposed traction field at the base of the lithosphere, derived from the mantle convection model (Supplementary Fig. 3), leads to the shearing and advection of materials underneath the North American lithosphere (Fig. 3). In our simulations, regions with a thickened crustal root develop weakening via conductive heating. Following this weakening, the extreme extension within zones of thick crustal welts results in free-boundary collapse of the thickened crust with substantial differential motion between brittle upper crust and weak lower crust, indicating lower crustal flow (Fig. 3). Based on our results, this extensional collapse is driven entirely by gravity acting on density differences created by topography of surface and Moho, along with internal density variations within the crust and upper mantle (Figs. 3 and 4).

As such, at the onset of the simulation (36 Ma), deformation is mainly concentrated in areas with high topography and thick crustal root. At this early stage of deformation, the model shows the formation of high angle (>38°) and high-strain-rate conjugate shear zones within the brittle upper crust that results in the formation of a necking zone there (Fig. 3a, g). The increase in local strain in the necking zone causes strain softening and significant plastic deformation in the upper crust (Fig. 3). Once necking has begun, it becomes the exclusive location of yielding and plastic deformation. After 2 Myr, the deformation is more concentrated at the high angle conjugate fault, and rheological contrast and body forces set up a shear zone and large offsets along the Moho (Figs. 3b, h and 4a). By 32.5 Ma, the right limb of the conjugated shear zone turns into a less-active normal fault, leading to the formation of an asymmetric shear zone with dominant slip along a low angle normal fault (Figs. 3c, i and 4b). Thinning of brittle crust below the highlands results in a reduction of the vertical load and consequently a horizontal pressure gradient at depth, which causes a lateral flow of lower crustal material toward the zone of necking, initiating doming of the brittle-ductile transition (Fig. 3b, c). As such, at ~32.5 Ma, the main detachment is at a shallower dip angle (20°), and the deformation is concentrated on this asymmetric shear zone (Fig. 4b). With ongoing lower crustal flow and crustal extension, the strain rate continues to localize strongly on the main detachment of the asymmetric fault zone. Through time, the dip of the main detachment gradually rotates to lower angles as it approaches the surface. By 30 Ma, the upper portion of the detachment fault located near the surface is at a low dip angle of 10°. This rotation of the detachment fault and formation of a MCC is consistent with the behavior of the “rolling hinge” mode^57^ in which the ductile middle crust and detachment fault are pushed upward by the local pressure gradient associated with necking zone within the upper crust (Figs. 3d, j and 4c). As a result, the middle crustal material on the footwall of the active fault is brought up to near sea level along the detachment fault, which is covered by the sediments. Finally, by 25 Ma, the middle crustal material on the footwall of the main detachment is located near the surface and the dip angle of the detachment fault is decreased to 8° (Figs. 3e, k and 4d). Following 25 Ma, strain rates on the shallower-dipping portions of the detachment diminish in magnitude in the direction of the dome peak, and the main detachment eventually turns into an inactive normal fault (Fig. 3f, l). The first phase of the crustal extension (36–20 Ma) results in the thinning of the ~60 km crustal root through a free-boundary collapse of a thickened crust, leading to a ~30 km crust. During the final phases of this thinning, extension is accommodated by more distributed faulting throughout the region, leading to a structural setting with basins and ranges at the end of the simulation in our model (Fig. 3f, l).

Our surface processes simulation shows that active sedimentation affects the strain localization and final crustal structure in our experiments, indicating that surface processes and sedimentation can to some extent control the development of lithospheric shear zones (Fig. 4). Our model shows that an asymmetric extensional depocenter (a supradetachment basin^58^) develops in the hanging wall of the active low-angle normal detachment fault (Fig. 4c). The subsidence of basin infill facilitates the formation and development of a MCC by increasing vertical transport of the weak lower-middle crustal materials into the shallower depths (Fig. 4c, d). Due to rapid uplift of the tectonically and erosionally denuded footwall, the basin fill is relatively thin (~0.5–2 km) (Fig. 4d and Supplementary Movie 1).

#### The symmetric conjugate fault mode (model 2)

**Fig. 5:**
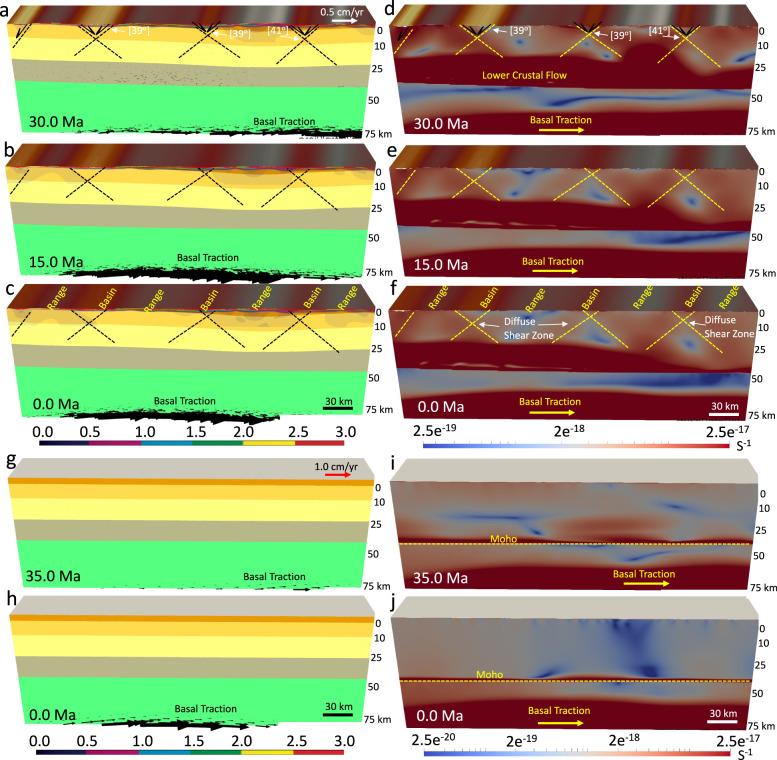
Deformation of the models with a flat Moho topography and a laterally varying surface elevation (non-uniform distribution of gravitational body forces) and a flat Moho topography and a flat surface elevation (uniform distribution of gravitational body forces) since the late Eocene under the influence of basal tractions and free slip side boundaries. **a**–**c** The evolution of crustal structure together with the magnitude of the plastic strain (failure) for the brittle upper crust at 30 Ma, 15 Ma, and end of the simulation at 0 Ma, respectively. Vectors represent the flow field through time. **d**–**f** The evolution of finite strain (values represent second invariant of strain tensor) magnitude at the brittle and ductile zones of the crust at 30 Ma, 15 Ma, and end of the simulation at 0 Ma, respectively. Note that in the experiment with a flat Moho topography and a laterally varying surface elevation the weakening and damage of the brittle upper crust and the resulting accumulated plastic strain and plastic shear zones produce basins and ranges. Necking centers in the crust remain active, and the conjugate shear zones are diffuse. Dips of the active shear zones remain high and no low-angle detachment fault or metamorphic core complex forms. **g**, **h** Similar to **a**–**c** but at 35 and 0 Ma, respectively. **i**, **j** Similar to **d**–**f** but at 35 and 0 Ma, respectively. Note that in the experiment with a uniform thickness crust there is no weakening and damage of the brittle upper crust in response to basal tractions at the base, free slip side boundary conditions, and uniform distribution of gravitational body forces. Hence, the crustal thickness experiences minimal defamation for the whole duration of our simulation. Experiments start at 36 Ma and evolve to 0 Ma. Stages of evolution are given in Ma.

The model with a paleo-topography, a flat Moho, and non-uniform distribution of GPE at the late Eocene demonstrates the formation of a symmetric conjugate fault mode. This model would be appropriate for a crustal root that had been removed and an initial topography that is supported dynamically. The constraint of sub-lithosphere tractions associated with our mantle convection model (Supplementary Fig. 3) along the base of the lithosphere results in an eastward basal flow. At the early stages of deformation (36–30 Ma), the model shows the lower crustal flow, topographic collapse, and formation of several pairs of high-angle (>39°) conjugate shear zones in the upper crust (Fig. 5a, b, d, e). Extension results in less concentration of strain in the shear zones (Fig. 5b, e) and no MCC mode forms. Through time, with further crustal extension, the model shows that the high-angle conjugate shear zones do not turn into asymmetric shear zones, since the conjugate shear zones are more diffuse in this simulation (Fig. 5b, e). With ongoing extension in the crust, active shear zones remain at high-angle dips (>39°), and a low-angle detachment fault does not form. The lithosphere evolution results in a structural setting of basins and ranges at the end of the simulation in our model (Fig. 5c, f and Supplementary Movie 2).

#### The rigid mode (model 3)

The model with a uniform thickness crust and a uniform distribution of GPE at the late Eocene demonstrates the formation of a rigid mode. This simulation shows that under the influences of a uniform distribution of GPE and sub-lithosphere traction field associated with eastward mantle flow along the base of the lithosphere following the slab rollback, the lower crustal flow associated with partial melting of the deep crust is not able to create sufficient shear localization in the crust to develop conjugated shear zones (Fig. 5g–j). As a result, after 36 Myr, the crust undergoes minor deformation, and the surface elevation does not change as well (Fig. 5g–j). This experiment explains why the cratonic part of the SWNA (e.g., the Colorado Plateau) underwent minor internal deformation following the onset of slab rollback at ~50 Ma, and during the Cenozoic extension phase within the BRP (Supplementary Movie 3).

### Body force and far-field extensional stress influences on lithosphere deformation and MCC formation (models 4–6)

Our simulations under the influences of different initial distributions of gravitational body forces along with constant far-field extensional stresses imposed on the left side of the finite element mesh show three end-member deformation modes that include: (1) the MCC mode, demonstrated by the formation of a low-angle detachment fault and exhumation of the ductile middle crust in a large domal upwarp, with later evolution to horst and graben topography; (2) the dome-like structure mode, demonstrated by the formation of conjugate faults and diapiric flow of the middle-lower crust without formation of a main detachment fault, also leading to a horst and graben topography; and (3) the pure-shear mode, demonstrated by the formation of diffuse shear zones and uniform extension of the crust, that leads to a horst and graben topography. Below we describe the main characteristics of these modes.

#### The MCC mode (model 4)

**Fig. 6:**
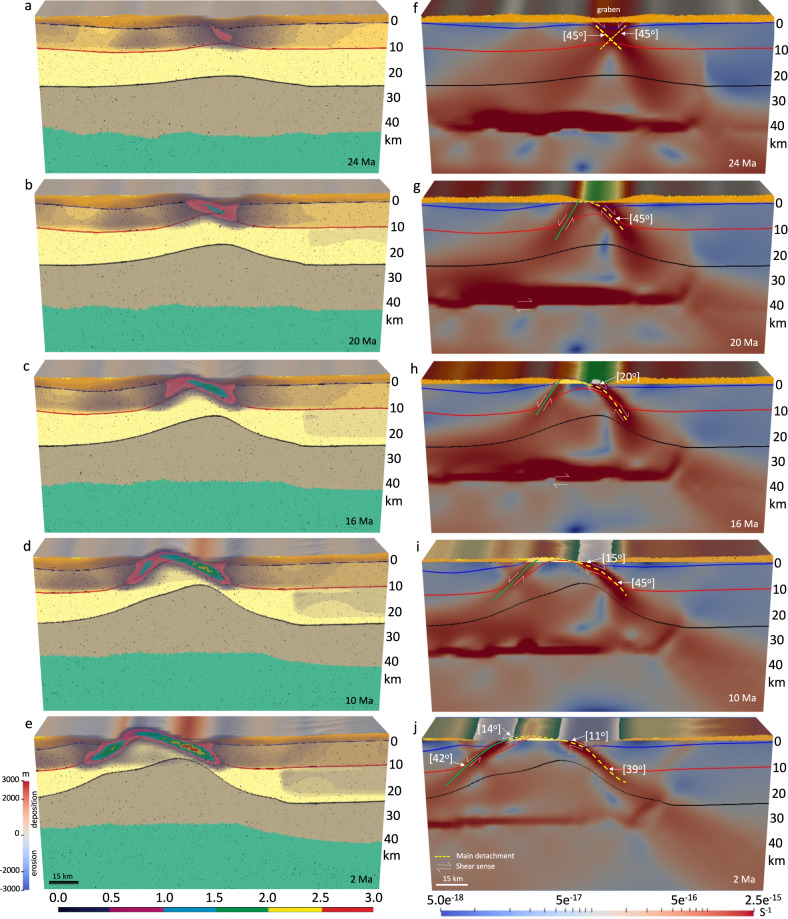
Deformation of the model with a crustal root and a laterally varying surface elevation since 30 Ma under the influence of left side velocity boundary condition, equal pressure at the base, and non-uniform distribution of gravitational body forces. **a**–**e** The evolution of crustal structure together with the magnitude of the finite plastic strain for the brittle upper crust at 24, 20, 16, 10, and 2 Ma, respectively. Cross-section (starting geometry) is at 38° N from ref. 37. **f**–**j** The evolution of finite strain (values represent second invariant of strain tensor) magnitude through the brittle and ductile zones of the crust at 24, 20, 16, 10, and 2 Ma, respectively. Strain develops in response to extension and fault rotation. Thicker dashed yellow line and green line denote where main and conjugate detachments, respectively, remain active and thinner dashed yellow line shows where detachment has become dormant. Note that in the experiment with a crustal root, a reduction in crustal thickness in response to isostatic rebound of the crustal root and collapse of topography increases the local strains within a necking zone that accommodates the doming and exhumation of the middle crust at the surface. Experiments start at 30 Ma and evolve to 0 Ma. Stages of evolution are given in Ma.

**Fig. 7:**
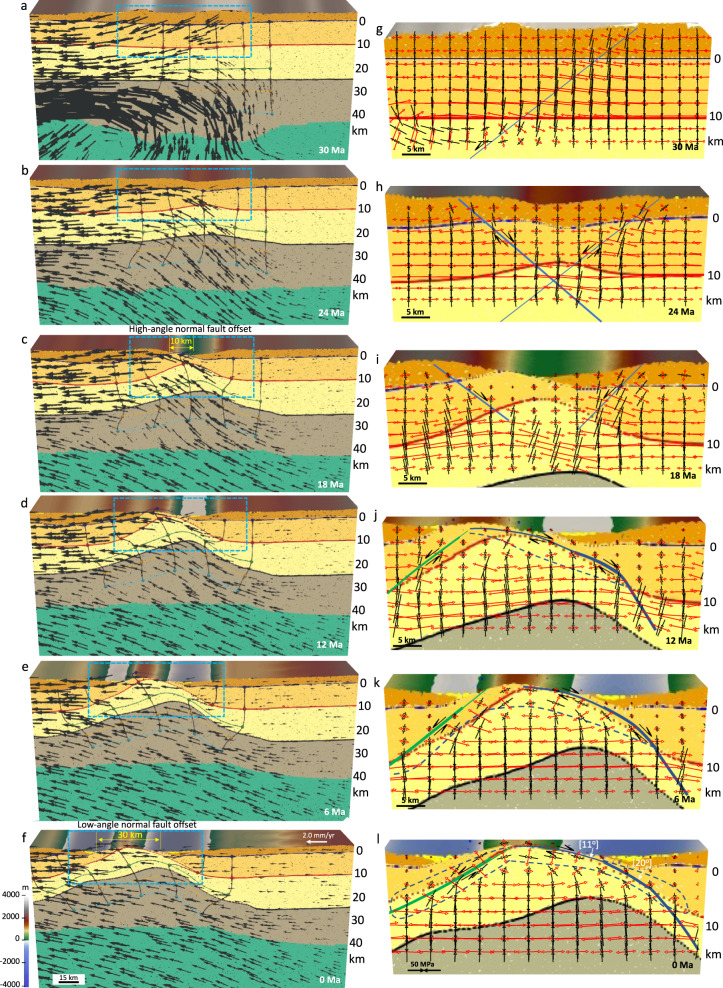
Within zones of high gravitational body forces, the relaxation of the crustal root generates a flow within the middle and lower crust. **a**–**f** Illustration of the interfacing of deformation between layers of the experiment with a crustal root at 30, 24, 18, 12, 6, and 0 Ma, respectively. Vectors represent the flow field through time. Blue dashed line represents the zoomed-in area for panels to the right. **g**–**l** Illustration of the rotation of principal axes of deviatoric stresses within the upper crust through high- and low-angle normal faulting. Red vectors represent tensional and black vectors represent compressional principal axes of deviatoric stresses. Black arrows show sense of slip offset accumulation on the detachment. Dashed line below detachment shows depth zone of mylonitic gneiss. Thicker blue and green lines denote where main and conjugate detachments, respectively, remain active and thinner blue and green lines show where detachment has become dormant. Note that the gravitational body forces generated by the topography and crustal root cause an upward flow pattern of the ductile lower and middle crust, which is facilitated by a dominant low-angle detachment surface. This detachment surface acquires large amounts of finite strain, consistent with thick mylonite zones found in metamorphic core complexes. Isostatic rebound exposes the detachment in a domed upwarp, while the final Moho geometry across the extended region relaxes to a flat geometry, in accord with seismic constraints. In total, there is a lateral extension of ~75 km so that ~40 km of extension is within the metamorphic core complex zone. Experiments start at 30 Ma and evolve to 0 Ma. Stages of evolution are given in Ma.

The model with high topography and a corresponding crustal root (non-uniform distribution of GPE), under the influence of a far-field extensional boundary condition, predicts vigorous upward ductile flow of the middle and lower crust and the formation of a MCC. As the Moho rebounds at the onset of deformation, the large magnitude of an upward motion of the lower crust and upper mantle sets up the viscous relaxation of the root (Figs. 6a–e and 7) and localizes strain in the upper crust below the highlands along a main detachment. This upward-directed flow results from mass conservation inside the domain and is a response to the constraint for pressure equilibrium at the base of the model. A conjugate shear zone on the left side (40°) accommodates counterclockwise rotation of the velocity field as it transitions from upward ascent, taken up along a main detachment system, to horizontal flow (Figs. 6f–j and 7).

The flattening of the crustal root results in a necking of the upper and middle crust and a reduction in local cross-sectional area of the crust which enhances localization of large amounts of strain below the highlands. The increase in local strains in the necking zone causes strain softening and significant plastic deformation in the upper crust (Fig. 6a, b). Once necking has begun, it becomes the exclusive location of yielding and plastic deformation. Concentrated shear transports the brittle-ductile transition upward along a doming detachment surface (Fig. 6a–e).

The opposite sense of shear along opposite dome limbs amplifies the doming within the MCC (Fig. 7d–f, j–l). As exhumation and doming of the brittle-ductile transition progresses, and a significant sedimentary basin forms at the surface enhancing loading, there is increased offset along the main detachment. This main detachment evolves from a high dip angle fault (45°) at depth to a low angle normal fault (15°) as it rolls into the shallower crustal zone. As the main detachment matures and acquires increasing finite strain, the conjugate high-angle detachment surfaces on both sides of the domal upwarp advect apart and the low-angle portion (11°) increases in length (Figs. 6f–j and 7g–l). Movement on the conjugate fault systems combined with doming accommodates the large horizontal offset of ~30 km from 20 to 0 Ma during this evolution. Strain rates are greatest on the steeply dipping (>30°) portions of the detachments while strain rates on the shallower-dipping (11–14°) portions of the detachment diminish in magnitude in the direction of the dome peak (Figs. 6h–j and 7f, l). The addition of modest (2 mm yr^−1^) velocity boundary conditions on the left side yields an upwarped detachment surface and exposed brittle-ductile transition that produces detachments on both sides of the domal upwarp with an opposite sense of vergence (direction of movement of upper plate) (Fig. 6j), whereas the model with gravity effect alone (model 1) generates a low angle normal fault with one dominant breakaway zone (Supplementary Movie 4). This model, therefore, can explain the opposite sense of vergence identified in the adjacent Cordilleran MCCs in NA, as defined by strike of mylonitic lineation^41^. For example, west-northwest vergence for the Ruby MCC (top-west shearing)^41^ and east-southeast vergence for the Snake Range MCC (top-east shearing)^41^.

#### The dome-like structure mode (model 5)

**Fig. 8:**
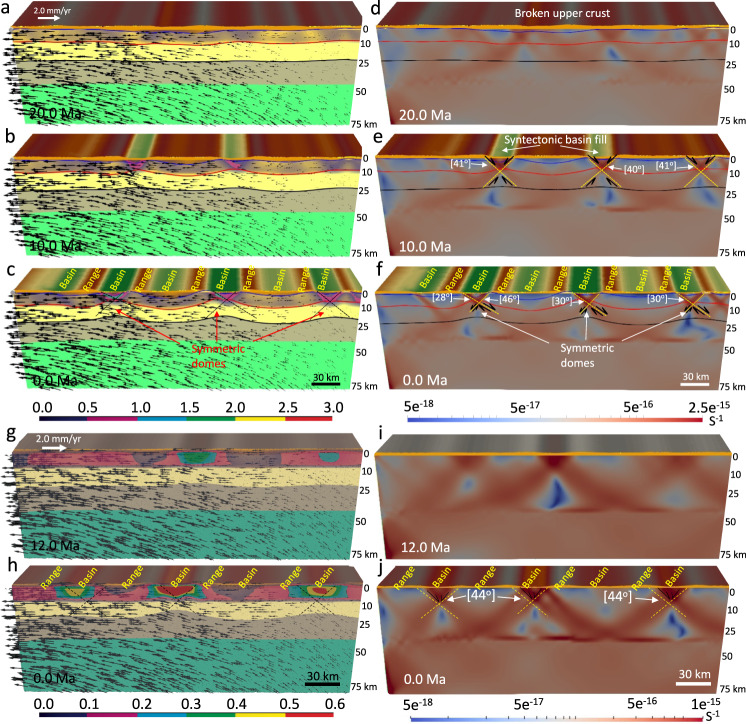
Deformation of the models with a flat Moho topography and a laterally varying surface elevation (non-uniform distribution of gravitational body forces) and a flat Moho topography and a flat surface elevation (uniform distribution of gravitational body forces) since 30 Ma under the influence of left side velocity boundary condition and equal pressure at the base. **a**–**c** The evolution of crustal structure together with the magnitude of the plastic strain (failure) for the brittle upper crust at 20 Ma, 10 Ma, and end of the simulation at 0 Ma, respectively. Vectors represent the flow field through time. **d**–**f** The evolution of finite strain (values represent second invariant of strain tensor) magnitude at the brittle and ductile zones of the crust at 20 Ma, 10 Ma, and end of the simulation at 0 Ma, respectively. Note that in the experiment with a flat Moho topography and a laterally varying surface elevation the weakening and damage of the brittle upper crust and the resulting accumulated plastic strain and plastic shear zones produce basins and ranges and symmetric dome-like structures in the crust. The right limb of active shear zones remain at high-angle dips and the left limb evolves into a low-angle detachment fault, but without metamorphic core complex formation. **g**, **h** Similar to **a**–**c** but at 12 and 0 Ma, respectively. **i**, **j** Similar to **d**–**f** but at 12 and 0 Ma, respectively. Note that in the experiment with a uniform thickness crust and flat Moho topography the weakening and damage of the brittle upper crust and the resulting accumulated plastic strain and plastic shear zones produce basins and ranges, but no doming or metamorphic core complex formation. Necking centers in the crust remain active, and the conjugate shear zones are diffuse or distributed throughout. Dips of the active shear zones remain high and no low-angle detachment fault forms. Experiments start at 30 Ma and evolve to 0 Ma. Stages of evolution are given in Ma.

The model with a paleo-topography, a flat Moho, and non-uniform distribution of GPE, under the influence of a far-field extensional boundary condition, demonstrates the formation of a symmetric dome-like structure mode. At the early stages of deformation (30–10 Ma), the model shows formation of several pairs of high-angle (>39°) conjugate shear zones and, hence, the formation of several necking zones in the upper crust (Fig. 8a, d). The extension results in less concentration of strain in the shear zones and no MCC mode forms. Through time, with further extension, topographic collapse, and erosion of the paleo-highlands, the model shows the formation of syntectonic sedimentary basins that cause an increase in concentration of strain rate into the high-angle conjugate shear zones. With further necking of the upper crust, the strain is more concentrated on the left limb of the conjugate shear zones, and the weak middle–lower crust slowly advects upward resulting in formation of symmetric domes, consistent with findings of Ma et al.^13^ (Fig. 8b, e). The opposite sense of shear along opposite dome limbs amplifies the doming. The right limb (>40°) of the conjugate shear zones accommodates counterclockwise rotation of the velocity field as it transitions from upward ascent to horizontal flow. With further extension in the crust and formation of sedimentary basins at the surface, enhancing loading, there is increased offset along a main detachment, and the shear zones turn slightly into asymmetric shear zones (Fig. 8c, f), in which the right limb of active shear zones remain at high-angle dips (>40°) and the left limb evolves into a low-angle detachment fault (28–30°). The lithosphere evolution results in a Basin and Range structural setting at the end of the simulation (Fig. 8c, f). This experiment shows that under the influence of extensional stresses and isothermal decompression of a weak and partially melted lower-middle crust, detachment-related domes^59^ can occur as a group in a large region of a paleo-highland system without the presence of a crustal root (dynamic support effect) (Supplementary Movie 5).

#### The pure-shear mode (model 6)

The model with a uniform thickness crust and uniform distribution of GPE, under the influence of a far-field extensional boundary condition, predicts formation of several diffuse shear zones transecting the crust, demonstrating the formation of a pure-shear mode (Fig. 8g–j). Although several necking zones form in the crust, there is not enough strain localization in the conjugated shear zones (Fig. 8g–j). Continued extension and distributed deformation in the crust result in ~5 km upward transport flow of the deep crust along shear zones which causes a uniformly thinned crust through this pure-shear mode (Fig. 8g–j). This experiment illustrates that the accommodation of extension within continental lithosphere under the influence of uniform distribution of GPE occurs in a pure-shear mode that involves uniform thinning of the crust and upper mantle through time, with preservation of a relatively flat Moho (Fig. 8g, i). At the later stages of deformation, the model shows the formation of high-strain diffuse shear zones in the crust, creating periodic relief (horsts) and basins (grabens) within the shallow crust, which is crisscrossed by high-angle (44°) conjugate normal fault structures (Fig. 8h, j). That is, without a weak zone to concentrate the extensional strain, extension is accommodated through the formation of ‘Basin and Range’ like structures instead of the formation of a MCC (Supplementary Movie 6).

## Discussion

**Fig. 9:**
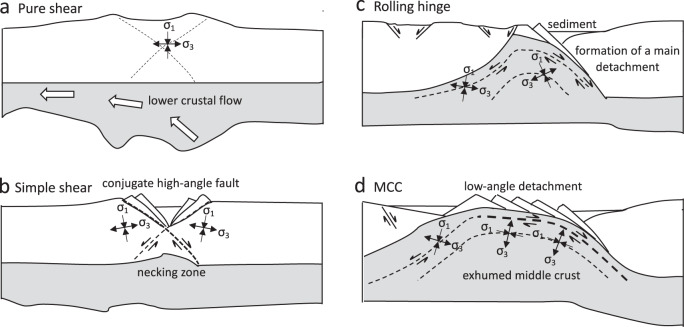
Schematics of the main mechanisms responsible for formation of the metamorphic core complexes at the lithospheric scale. **a** Within continental crust, zones of high gravitational body forces are highly affected by lower crustal flow which causes pure shear and topographic collapse. **b** Development of a simple shear phase associated with crustal root rebound and topographic collapse, together with the formation of a necking zone and high-angle conjugate fault. The counterclockwise rotation of the velocity field, as it transitions from upward ascent to horizontal flow, is accommodated along a main detachment surface and a nearby conjugate detachment. **c** The relaxation of the crustal root generates an upward transport and doming of middle crust that is primarily accommodated along the main detachment zone (the rolling hinge phase). **d** A final configuration of the metamorphic core complex involving an exhumed low-angle detachment within the domal region. Black arrows represent principal axes of stresses on the conjugate fault and detachment surface. MCC metamorphic core complex.

Our models show the primary physical conditions for development of MCCs at the crustal and lithosphere scales, together with their patterns of deformation at the crustal scale. Models 1 and 4, that include highlands and a crustal root (non-uniform distribution of GPE), demonstrate that MCCs develop out of a long-lived upward crustal flow driven by gravitational body forces in both (1) a basal traction (stress) field boundary setting associated with mantle convection, but with a free slip side boundary condition or (2) a far-field extensional stress boundary setting, respectively. The isothermal decompression of the hot deep crust results in flattening of the Moho through time (Fig. 9a, b). Strain in the middle-lower crust is accommodated by distributed flow because it does not reach the yield stress limit or the point of strain softening. Under the influence of our two different boundary condition settings, mentioned above, a counterclockwise flow is established with the middle and lower crust moving up and then flowing towards the boundary that is moving or collapsing away. This produces a dome-shaped structure that moves the ductile middle-lower crust upward towards the surface (Fig. 9c, d). The development of the MCC is supported by the flow of the hot and weak lower crust that feeds the exhuming dome. This appears to explain the belt of MCCs in Nevada and, in general, central-northern section of the Cordillera of NA (Fig. 1a), where most kinematic indicators show an upper plate movement toward the east^41^, involving a main detachment that projects beneath the Colorado Plateau (on right side of model). The key is the presence of the crustal root and the flow pattern set up by its rebound and boundary conditions. The final relaxation of the root, the flattening of the Moho, and the collapse of higher topography lead to a more even body force distribution (or uniform distribution of GPE), which heralds the “Basin and Range” phase of distributed extension.

Our MCC modes (models 1 and 4), therefore, address the kinematics, timing, and formation mechanism of MCCs in the Cordillera of NA^41^ (e.g., Raft River^60^, Snake Range^54^, and Buckskin^41^ MCCs) by simultaneously incorporating the dynamic influences within the lithosphere based on changes in gravitational body forces, thermal and rheological variations, as well as deeper mantle dynamics effects. Based on our results, the crustal extension within areas with thickened crustal welts and high elevations in SWNA was facilitated by rheological weakening of the middle-lower crust via conductive heating and partial melting of the deep crust following the lithosphere foundering of the Laramide flat subduction (Figs. 3, 4, 6, and 7). Spencer and Reynolds^52^ suggested that MCC formation in western Arizona was facilitated by a buoyant crustal root. However, more recent work has suggested that this root in western Arizona might have been removed prior to MCC formation via low-angle subduction during the Laramide^61,62^. Spencer et al.^61^ argued that following the postulated root removal in western Arizona, the lower crustal viscosities within the thickened crust would have been low and therefore the crust could have been highly mobile there. This weak and highly mobile lower crust is consistent with our findings (models 1 and 4) and is a necessary component for facilitating the upward transport of ductile middle crust along the dominant detachment where strains get concentrated.

Hence, our simulations provide constraints on the landscape evolution and MCC formation in SWNA. The presence of the felsic magmatism from northeast to southwest Nevada^37,63^ following slab rollback, during the late Eocene to 10 Ma, reveals high temperature and partial melting of the lower crust beneath and adjacent to the highlands^64,65^. Such partial melting of the lower crust, as argued by other studies^23,24^, could have made the average viscosity of the lower crust quite low (Fig. 2a) and facilitated the lower crustal flow during the initial phase of topographic collapse. Our numerical modeling of MCCs, therefore, show that extensional collapse of highlands with a thickened and anomalously hot crustal root depicts the first phase of extensional collapse in SWNA from the onset of the slab rollback at the late Eocene to ~10 Ma, leading to MCC formation. In the final phases of MCC formation, there are no longer concentrated body forces in the MCC region. This explains why the later phases of extension, with the flat Moho, evolved into a more widely distributed zone of extension. Analogous to our experiment with a flat Moho, this later phase of extension represents the Basin and Range period of faulting from ~10 Ma to present.

Based on our results, the presence of high gravitational body forces in SWNA, associated with a thickened crustal welt, should have been the major factor guiding topographic collapse, crustal extension, and development of the main detachment fault and mylonite zone that formed the MCCs from the late Eocene to ~10 Ma. This major result confirms the importance of surface and Moho topography and internal density variations throughout the lithosphere in driving the early extensional history. Therefore, our results demonstrate that tractions associated with the mantle convection effect following the slab rollback played a much lesser role in their influence on the stress field than do the GPE gradients, and that slab rollback only promoted the timing of extensional collapse of a thickened crust in SWNA by causing rheological weakening via heating and partial melting of the deep crust. However, in the absence of a crustal root, we argue here that the partial melting of the deep crust alone was not sufficient to produce MCCs.

The overarching conclusion of this study is that belts of MCCs are a fossil signature of collapsed highlands, and they develop in continental crust that has become thickened and thermally softened, and that under the influence of a far-field extensional stress conditions, more symmetric distributed extensional structures develop in continental places without existence of highlands and crustal roots (with nearly uniform distribution of GPE). This conclusion for the Cordillera of NA can be strongly supported by a variety of proxies that have been employed by many studies to estimate paleo-crustal thickness, paleo-topography, and paleo-altitude across the region.

For example, recent isotope analyses and geochemical evidence^66,67^ support the presence of an orogenic highland with a ~60 km-thick crust and an associated paleo-elevation of ~3 km in SWNA along the edge of the Colorado Plateau during the Laramide and before the onset of the slab rollback. Since the isotopic composition of precipitation somewhat directly scales with elevation, it can be used to reconstruct topographic histories of mountain belts^68^. The δD measurements in hydrated glass from ignimbrites^69^ indicate the presence of a high, broad orogen that stretched across northern to southern Nevada during Eocene to Oligocene, with the highest elevations of ~3.5 km in the late Oligocene. Gébelin et al.^70^ results based on δ^18^O and δD values calculated from muscovite in Snake Range Mountain (northwestern Nevada) yield an elevation of ~3.8 km between 27 and 20 Ma. Low Oligocene-Miocene d^18^O and dD values of meteoric water from the Snake Range MCC reflect that precipitation was sourced at high elevation^70^. Stable isotope data from the Raft River detachment shear zone in Utah indicate that very low δD surface-derived fluids penetrated the brittle–ductile transition as early as the mid-Eocene during a first phase of exhumation along a detachment rooted to the east^71^. The paleo-botanical study by the authors in ref. 72 suggests the presence of an Eocene-Oligocene highland of ~4 km or more in the northeast Nevada. Paleo-botanical results for late Eocene House Range flora in Sevier Desert (west-central Utah) yield a paleo-elevation of ~3–4 km at ~31 Ma^73^. Paleo-botanical analyses obtained by the authors in ref. 74 for several mid-Miocene floras in eastern Nevada suggest a paleo-altitude of ~3 km at about 15–16 Ma. Their assumption that the highlands had collapsed by ~13 Ma agrees with our conclusion that the second phase of extension for the BRP in SWNA followed the topographic collapse, and that the result of extension on a uniformly distribution of GPE (model 6) is the formation of high-angle conjugate faults that lead to formation of basins and ranges.

While Mix et al.^68^ suggest that slab rollback may have been the primary source of a wave of uplift of the Nevadaplano (~2.5 km) that swept from north to south during the Oligocene and before the topographic collapse, our models 2 and 5, with a laterally varying surface elevation and a flat Moho (without a crustal root), under the influences of both basal tractions from the mantle convection and far-field extensional stresses do not result in formation of MCCs. In the absence of a crustal root, model 5 shows formation of dome-like structures in which ductile middle crust flows laterally and bulges upwards at the extending brittle upper crust and eventually freezes into place within the upper crust. Therefore, our results demonstrate that the MCC formation in the Cordillera of NA requires paleo-elevations that are expected to have still been high (>3 km) above the substantial crustal welt that was present just prior to the slab rollback.

Moreover, the results in this paper provide resolution on the controversy regarding whether the detachment faults originate at a low-angle, as observed in field exposures^48,75^, or whether they accommodate most or all slip at high-angles^17,18,31^. Our simulations show that, through the topographic collapse, detachment faults initiate as steep faults, and these steep faults remain active during much of the MCC evolution. However, the main detachment shallows near the surface as the domal upwarp grows. The dip of the zone of finite plastic strain (mylonite zone) also shallows as the main detachment evolves. The advanced stages of the MCC geometry involve a low-angle detachment surface that exposes the brittle-ductile transition. The steep portion of the main detachment possesses the highest strain rates throughout the MCC evolution. As this detachment warps and acquires a shallower dip at shallower crustal depths, strain rates diminish in magnitude and reach zero toward the dome peak. Rapid slip rates along the steeply dipping portion of the main detachment in model 4 agrees with the abundant global observations of steep dip angles for normal fault earthquakes within extensional settings with very few examples of low-angle normal fault mechanisms^76^. However, our model shows stress rotations within the shallow crust that allow for limited slip along the detachment where it has rotated into the lower dip angles. Recently, surface rupture was documented on shallow-dipping normal faults during the El Mayor–Cucupah earthquake^77^. There, rupture occurred primarily on steeply dipping segments of a complex fault geometry at depth, but slip was diverted near the surface to rupture along low-angle normal fault segments. While uncommon, such slip on the shallow crustal, low-angle portions of the detachment may explain the field observations of pseudotachylytes within some shallow dipping detachment zones. Our model results thus explain the seemingly contradictory interpretations that low-angle normal faults show evidence of slip while at low angles^75^, versus the recent interpretation that slip on the main detachment occurs primarily at high angles^78^.

The modeling described here can also be applied to other MCCs around the world where post-thickening extension appears applicable. Some examples where the collapse of a zone of crustal thickening and topography are likely the primary factor for MCC development include the areas north of the BRP^41^, including the Canadian Cordillera^79^, the eastern Mediterranean region and central Anatolia^80^, the northern Apennines^81^, the Alboran Sea and Gibralter Arc^82^, and Papua New Guinea^83^. Finally, the collapse of highlands and relaxation of a crustal root under extensional settings can likely explain many exposures of ancient gneissic domes around the world^84^, where the brittle cover has likely been removed through erosion, exposing the core of the uplifted metamorphic dome.

## Methods

### Numerical experiment set-up, boundary conditions, and solutions for lithosphere thermomechanical simulation

We develop time-dependent 2.5-D coupled −thermal and mechanical tectonic and geodynamic models of the lithosphere (crust and upper mantle) using Underworld Geodynamics (UWGeodynamics) code^85–88^. UWGeodynamics accounts for conservation of mass, energy, and momentum, and utilizes a Lagrangian integration Particle-In-Cell Finite-Element-Method approach (tracking particles embedded in the deforming material relative to the mesh) for the solutions to Stokes flow-type configurations and heat transport equations. Fundamental UWGeodynamics equations for conservation of mass, energy, and momentum include:

1 $$\nabla \cdot u=0,$$

2 $$\rho {C}_{{{{{{\rm{p}}}}}}}\left(\frac{\delta T}{\delta t}+u\bullet \nabla T\right)=\nabla \bullet \left(k\nabla T\right)+Q,$$

3 $$\nabla \bullet \left(\eta \nabla u\right)-\,\nabla {{{{{\rm{p}}}}}}=-\rho g,$$

where *u* is velocity, ${T}$ is temperature, $t$ is time, ${C}_{{{{{{\rm{p}}}}}}}$ is specific heat capacity, $\rho$ is density, $k$ is thermal conductivity, $Q$ is an additional heat source for the energy equation, $\nabla u$ is the velocity gradient, $\nabla {{{{{\rm{p}}}}}}$ is the pressure gradient, $\eta$ is the viscosity, $\rho g$ is the driving force, and $\nabla \bullet (\eta \nabla u)$ is the stress gradient.

In our simulations, we incorporate additional geodynamic terms like radiogenic heating ($H$) into the Eq. (2) for conservation of energy as:

4 $$H=\frac{{R}_{{{{{{\rm{h}}}}}}}}{\rho {C}_{{{{{{\rm{p}}}}}}}},$$

where ${R}_{{{{{{\rm{h}}}}}}}$ is the rate of radiogenic heat production. $H$ specifies the magnitude of energy that is added into the system. Therefore, in our simulations, the rate of change of temperature as a function of heat diffusion (${\alpha \nabla }^{2}T$), heat advection ($u\bullet \nabla T$), and radiogenic heat production ($H$) is expressed as:

5 $$\frac{\delta T}{\delta t}={\alpha \nabla }^{2}T-u\bullet \nabla T+H,$$

where $\alpha$ is the thermal diffusivity $(\alpha=\frac{k}{\rho {C}_{{{{{{\rm{p}}}}}}}})$. Equation (5) ensures conservation of energy and allows the coupling of densities and viscosities to temperature.

The experimental domain is composed of a finite-element mesh that is 420 km long and 90 km thick (Fig. 2). Experiments have a 0.5 km computational grid resolution from the top of the model (15 km above sea-level) to 75 km depth. Experiment structures are as follows: For experiments 1–3, we apply time-dependent stress boundary condition from the mantle convection simulation to the base of the finite element mesh that includes (1) a reconstructed crustal structure at the late Eocene from the model of ref. 37, (2) a reconstructed surface elevation at the late Eocene from the model of ref. 37 and a flat Moho boundary at 45 km depth, and (3) a uniform thickness crust with a surface elevation of 4 km and a Moho boundary of 45 km depth. We apply a free slip boundary condition to the top, left, and right sides of the finite element mesh.

For experiments 4–6, we apply uniform stretching (2.0 mm yr^−1^) to the left side of the finite element mesh that includes: (1) a reconstructed crustal structure at the late Eocene from the model of ref. 37, (2) a reconstructed surface elevation at the late Eocene from the model of ref. 37 and a flat Moho boundary at 45 km depth, and (3) a uniform thickness crust with a surface elevation of 4 km and a Moho boundary of 45 km depth. We apply a free-slip boundary condition at the right and top of models 4–6 and an isostatic pressure equilibrium is applied as a boundary condition at the base of the models (75 km). This isostatic function at the base of the model in simulations 4–6 allows inflow of material to balance the extension-driven outward flow of material at the left side of the model. The implementation of isostatic equilibrium at the base of the model in our simulations is done by means of a function that calculates the local Pratt isostasy at each Eulerian node at the base of the model and maintains the constant pressure by applying the necessary upwards velocity. This basal velocity boundary condition is expressed based on a Dirichlet condition using the density-weighted velocity to adjust the basal velocities through time^89,90^. The isostatic function at the base of the model therefore allows inflow of material to balance the extension-driven outward flow of material at the left side of the model and provides a way to maintain the mass inside the domain through updating the velocity field in order to simulate the isostasy.

All simulations (models 1–6) include shallow crust at 10 km depth below sea level (2720 kg m^−3^), middle crust from 10 to 25 km depth (2720 kg m^−3^), deep crust from 25 to Moho (45–65 km) (2720 kg m^−3^), lithospheric mantle from Moho (45–65 km) to 75 km depth (3370 kg m^−3^). A swarm of passive markers is used to represent the finite strain field within the crust (Fig. 2a). The progressive deformation of these markers allows finite strain intensity and orientation to be tracked across the suite of experiments. We assign specific properties listed in Supplementary Table 1 to each material included in our models.

We also solve for the thermal evolution of the model through time. The simulation is initiated with a temperature field that is derived from solving a transient coupled thermomechanical model wherein the velocity boundary conditions at the left and right of the model are set to 0 mm yr^−1^. The initial temperature field for the model is defined based on a linear initial geotherm^91^ with a thermal boundary condition at the top of the model that is the absolute temperatures of 273 °K, a laterally varying temperature field at the base of the model from the model of ref. 40, and a constant basal heat flow of 0.022 W m^−2^ together with zero heat flow across the lateral sides of the model. To simulate the effect of slab rollback and temperature variations at the base of the lithosphere, the starting temperature boundary condition at the base of the model is updated using a time-dependent thermal boundary condition that is defined based on absolute temperatures at the base of the model that account for temperature changes from the model of ref. 40. The model of ref. 40 was based on the reconstructed (in position) magmatism as a proxy for centers of temperature perturbations over time^37^ and these patterns of volcanic centers are assumed to reflect slab rollback history.

In addition to solving the thermal evolution of the model, we also solve the equilibrium equations for viscous–plastic flow in two dimensions. Therefore, in our simulations material deformation is expressed based on non-Newtonian visco-plastic rheologies with viscosity dependent on temperature, pressure, and strain rate (including a strong, viscous/frictional-plastic upper crust together with a weak, visco-plastic middle and lower crust). In the upper crust strain accommodation occurs by plastic shear zones while in the lower crust viscous flow and plasticity compete. The implementation of lithospheric deformation in terms of a visco-plastic rheology is incorporated by decomposing the deviatoric strain rate into viscous and plastic components that are solved sequentially. The flow is computed through dislocation creep (*n* > 1 and *G* = 0, where *n* is the power law stress exponent and *G* is the grain size exponent)^92,93^ for the viscous component, which can be expressed using the following equation:

6 $${\eta }_{{{{{{{\rm{eff}}}}}}}}^{{{{{{{\rm{viscreep}}}}}}}}(T,P,\dot{\epsilon })=f{A}^{\frac{-1}{n}}{\dot{\epsilon }}^{\frac{\left(1-n\right)}{n}}{{\exp }}\left(\frac{E+{PV}}{{nRT}}\right),$$

where *A* is a pre-exponential factor that is not sensitive to thermochemical conditions, *n* is the stress exponent and is a non-dimensional constant, $\dot{\epsilon }$ is the strain rate, *E* is the activation energy, *P* is the pressure, *V* is the activation volume, *R* is the gas constant, *T* is the temperature, and *f* is a scaling factor chosen to represent materials that are viscously weaker or stronger than the reference flow law. In our simulations when the state of stress is below the frictional-plastic yield, the flow is viscous and is specified by temperature-dependent non-linear power-law rheologies based on laboratory measurements for dislocation creep on wet quartz for the crust and dry olivine for the mantle. Power law creep parameters applied in our thermomechanical model are listed in Supplementary Table 2 and are taken from ref. 24. The model with a crustal root includes a stronger middle and lower crust for the Colorado Plateau area (*f* = 20).

For the plastic component of the flow (frictional-plastic yielding), failure is determined using a pressure-dependent Drucker–Prager yield criterion, which is equivalent to the Mohr–Coulomb yield surface for incompressible deformation^93^:

7 $${\sigma }_{{{{{{{\rm{yield}}}}}}}}={({J}_{2}^{/})}^{1/2}={Ap}+B={\sin }{{\varnothing }}\,p+C\,{\cos }{{\varnothing }},$$

where ${J}_{2}^{/}$= $\frac{1}{2}$ ${\sigma }_{{ij}}^{/}{\sigma }_{{ij}}^{/}$ is the second invariant of the deviatoric stress tensor, ${\sigma }_{{ij}}^{/}$ is the deviatoric stress tensor, *C* is the cohesion, $\varnothing$ is the internal angle of friction, and *p* is the pressure. In the crust and mantle, frictional sliding is modeled via Mohr Coulomb criterion. In our simulations, the brittle properties of materials change by local strain accumulation so that both cohesion and friction coefficient decrease linearly with strain. Models 4–6 include a starting cohesion of 15 MPa and a coefficient of friction of 0.44^23^. During frictional strain softening, the friction coefficient ($\mu$) reduces linearly from 0.44 to 0.088 for brittle strain between 0.0 and 0.5. Models 1–3 include a starting cohesion of 5 MPa and a coefficient of friction of 0.3. During frictional strain softening, the friction coefficient ($\mu$) reduces linearly from 0.3 to 0.03 for brittle strain between 0.0 and 0.5.

Therefore, under lower pressure and high stress conditions, when differential stresses reach the yield stress, the material fails, and deformation is modeled by an effective viscosity as:

8 $${\eta }_{{{{{{{\rm{eff}}}}}}}}^{{{{{{{\rm{plastic}}}}}}}}=\frac{{\sigma }_{{{{{{{\rm{yield}}}}}}}}(p,\dot{\epsilon },\epsilon )}{2\dot{\epsilon }},$$

where $\dot{\epsilon }={(\frac{1}{2}{\dot{\epsilon }}_{{ij}}{\dot{\epsilon }}_{{ij}})}^{1/2}$ is the second invariant of the strain rate tensor. As strain is accumulated, yielding rheologies linearly interpolate between their original values (e.g., cohesion, friction coefficient) to their damaged values. we define two viscosity thresholds of ${\eta }_{{{{{{\rm{min}}}}}}}=$ 1e^18^ and ${\eta }_{{\max }}=$ 5e^23^. Hence, ${\eta }_{{\min }}\le {\eta }_{{{{{{{\rm{eff}}}}}}}}\le {\eta }_{{\max }}.$

In our simulations, the relation between temperature and density is expressed as:

9 $$\rho \left(T\right)={\rho }_{0}\left[1-\beta \left(T-{T}_{0}\right)\right],$$

where ${\rho }_{0}$ is the reference density, $\beta$ is the coefficient of thermal expansion, $T$ is the temperature, and ${T}_{0}$ is the reference temperature. We assume a coefficient of thermal expansion of 2.8 × 10^−5^ k^−1^ in the crust and mantle.

### Computation of the partial melting of the deep crust for the thermomechanical model of the lithosphere

To account for partial melting of the deep crust, in addition to incorporating radiogenic heating ($H$), we also incorporate thermal aspects of partial melting ($F$) into the Eq. (2) for conservation of energy as:

10 $$F=-1\times \left(\frac{{L}_{{{{{{\rm{f}}}}}}}}{{C}_{{{{{{\rm{p}}}}}}}}\right)\left(\frac{{\delta M}_{{{{{{\rm{f}}}}}}}}{\delta t}\right),$$

where ${L}_{{{{{{\rm{f}}}}}}}$ is the latent heat of fusion that represents the amount of energy consumed during a phase change from solid to liquid, and ${M}_{{{{{{\rm{f}}}}}}}$ is the melt fraction. Therefore, in our simulations, the rate of change of temperature as a function of heat diffusion (${\alpha \nabla }^{2}T$), heat advection ($u\bullet \nabla T$), radiogenic heat production ($H$), and heat changes associated with partial melting processes (${FT}$) is expressed as:

11 $$\frac{\delta T}{\delta t}={\alpha \nabla }^{2}T-u\bullet \nabla T\,+H+{FT}.$$

In our simulations, the mechanical effect associated with partial melting of the lower crust is determined by reduction of the viscosity of the lower crust within a melt range of 0.15 to 0.3. Melting is applied to an existing viscous rheology, and is calculated as:

12 $${M}_{{{{{{{\rm{int}}}}}}}}=1+\left(\frac{{M}_{{{{{{\rm{f}}}}}}}-\,{L}_{{{{{{\rm{f}}}}}}}}{{L}_{{{{{{\rm{f}}}}}}}-\,{U}_{{{{{{\rm{f}}}}}}}}\right),$$

13 $${\eta }_{{{{{{\rm{m}}}}}}}=\eta \times ({1+M}_{{{{{{{\rm{int}}}}}}}}+{\eta }_{{{{{{\rm{f}}}}}}}\times (1-{M}_{{{{{{{\rm{int}}}}}}}})),$$

where ${\eta }_{{{{{{\rm{m}}}}}}}$ is the updated viscosity after material melts, $\eta$ is the viscous rheology, and ${M}_{{{{{{{\rm{int}}}}}}}}$ is a normalized linear interpolation of the percentage of the melt fraction (${M}_{{{{{{\rm{f}}}}}}}$) between the upper (${U}_{{{{{{\rm{f}}}}}}}=$ 30%) and lower (${L}_{{{{{{\rm{f}}}}}}}$ = 15%) limit of the melt fraction range, and ${\eta }_{{{{{{\rm{f}}}}}}}$ is the melt viscous softening factor that lower crust material undergoes once melted (Supplementary Table 3). When the melt fraction increases from 15 to 30%^94,95^, the viscosity decreases by 2 orders of magnitude consistent with findings of ref. 40 who argued that timing of extensional collapse of paleo-highlands in SWNA required 2 orders of magnitude weakening of effective viscosities of lithosphere. Therefore, when the melt fraction is 15%, the viscosity of the melted crust is that of the non-melted surroundings, and when the melt fraction is 30%, the viscosity of the melted crust is 100 times lower than in surrounding material. It is important to mention that segregation of the melt from the host rock does not happen in our simulations and melt phase remains in its source, which is consistent with observations of migmatite-cored metamorphic core complexes in which a relatively small volume of leucogranite is extracted from the partial melt layer^90,96^.

The melt fraction (${M}_{{{{{{\rm{f}}}}}}}$) in our simulations is a function of the super-solidus dimensionless temperature^97^ and is calculated as:

14 $${T}_{{{{{{{\rm{ss}}}}}}}}=\frac{\left(T-\left({{T}_{{{{{{\rm{s}}}}}}}+{T}}_{{{{{{\rm{l}}}}}}}\right)\,{{\times }}\,0.5\right)}{\left({T}_{{{{{{\rm{l}}}}}}}-{T}_{{{{{{\rm{s}}}}}}}\right)},$$

15 $${M}_{{{{{{\rm{f}}}}}}}=0.5+{T}_{{{{{{\rm{ss}}}}}}}+({{T}_{{{{{{\rm{ss}}}}}}}}^{2}-0.25)\times (0.4256+2.988\times {T}_{{{{{{\rm{ss}}}}}}}),$$

where *T*_ss_ is the super-solidus, *T*_s_ is the solidus temperature, and *T*_l_ is the liquidus temperature. The solidus and liquidus for the crust and mantle are both temperature- and pressure-dependent and are parameterized by a polynomial relationship between temperature and pressure^97^ as:

16 $${T}_{{{{{{\rm{s}}}}}}}={a}_{{{{{{\rm{s}}}}}}}+{b}_{{{{{{\rm{s}}}}}}}P+{c}_{{{{{{\rm{s}}}}}}}{P}^{2},$$

17 $${T}_{{{{{{\rm{l}}}}}}}={a}_{{{{{{\rm{l}}}}}}}+{b}_{{{{{{\rm{l}}}}}}}P+{c}_{{{{{{\rm{l}}}}}}}{P}^{2},$$

where *a*, *b*, and *c* are constants and are defined in Supplementary Table 3. These parameters for calculating melt fraction were derived from refs. 98, 90. As such, our final estimate of material density is dependent on melt fraction and temperature. Therefore, the density change caused by melt fraction expansion factor $(\gamma=0.13)$^90^ and the fraction of melt (${M}_{{{{{{\rm{f}}}}}}}$) affects the final density evolution of materials in our simulations which is expressed as:

18 $$\rho \left(T,F\right)={\rho }_{0}\left[1-\left(\beta \delta T\right)\,-\left(\gamma F\right)\right].$$

### Mantle convection simulation

We use the open-source finite-element code ASPECT^99–101^ (short for Advanced Solver for Problems in Earth ConvecTion) to solve the equations for conservation of momentum, energy, and mass, assuming incompressible Stokes flow and the extended Boussinesq approximation:

19 $$\nabla \cdot u=0,$$

20 $$\rho {C}_{{{{{{\rm{p}}}}}}}\left(\frac{\partial T}{\partial t}+u\bullet \nabla T\right)=\nabla \bullet \left(k\nabla T\right),$$

21 $$\nabla \bullet \left(2\eta \dot{\epsilon }\right)-\,\nabla p=\,-\rho g,$$

where *u* is velocity, ${T}$ is temperature, $t$ is time, $\rho$ is density, $\rho={\rho }_{0}[1-\alpha \left(T-{T}_{0}\right)]$ with ${T}_{0}$ the reference temperature (1600 °K), ${\rho }_{0}$ the reference density (3300 kg m^−3^), and $\alpha$ the coefficient of thermal expansion (3 e^−5^ °K^−1^), ${C}_{{{{{{\rm{p}}}}}}}$ is specific heat capacity (1250 J °K^−1^ Kg^−1^), $k$ is thermal conductivity (0 W m^−1^ K^−1^), $\eta$ is the viscosity, $\dot{\epsilon }$ is the deviator of the strain rate tensor, $\dot{\epsilon }=\frac{1}{2}(\nabla u+{(\nabla u)}^{T})$, $\nabla \bullet (2\eta \dot{\epsilon })$ is the stress gradient, $\nabla p$ is the pressure gradient, and $\rho g$ is the driving force.

The domain of our global mantle convection simulation is a 3-D spherical shell. In our simulations, we use an “initial global refinement” parameter of 4 so that the finite-element mesh contains 12 × (32)^3^ = 393,216 cells. We apply free-slip boundary conditions at the surface and core–mantle boundary, and we remove the net rotation component of the flow solution. Using the global density distribution model TX2008^55,56^ we simulate the temporal evolution of global mantle flow by backward advecting density perturbations, assuming no diffusion. Present-day temperatures are obtained from the TX2008 density field through thermal expansion. Boundary temperatures are 1600 and 3300 °K at the surface and core-mantle boundary, respectively. Our convection simulation incorporates Newtonian rheology with a 1-D viscosity profile (V2) from ref. 55. We determine estimates of spatial and temporal variations in deviatoric stress tensors and the associated traction field at different depths from the time-dependent mantle convection model.

### Surface processes simulation

While the thermomechanical model is 2-D, by extrapolating the velocity field at the surface in the third dimension we produce a 2.5-D model. This model is a two-way coupled thermomechanical model with surface processes, where the velocity field retrieved from the thermomechanical model is used to advect the surface in the surface processes model. The surface is subjected to erosion and deposition. The distribution of materials in the thermomechanical model is then updated after surface processes simulation is completed at each time step. We use the open‐source landscape evolution modeling code Badlands^102^ to simulate the evolution of topography, sediment erosion, transport, and deposition through time.

In Badlands, the continuity of mass is defined by:

22 $$\frac{{{{{{{\rm{d}}}}}}Z}}{{{{{{{\rm{d}}}}}}t}}=-\nabla \bullet {q}_{{{{{{\rm{s}}}}}}}+u,$$

where $u$ is the uplift rate (m yr^−1^) and ${q}_{s}$ is downhill sediment transport per unit width (m^2^ yr^−1^). The downhill sediment transport (${q}_{{{{{{\rm{s}}}}}}}$) can be represented as a combination of incorporating sediment transport by (1) channel flow $({q}_{r})$, which is described by a stream power-law, and (2) long-term slope-driven diffusive processes $({q}_{{{{{{\rm{d}}}}}}})$, described by simple creep^103^ as:

23 $$-\nabla \bullet {q}_{{{{{{\rm{r}}}}}}}\,=-{k}_{{{{{{\rm{d}}}}}}}{\left({PA}\right)}^{m}{\left(\nabla Z\right)}^{n},$$

24 $$-\nabla \bullet {q}_{{{{{{\rm{d}}}}}}}=-{k}_{{hl}}{\nabla }^{2}Z,$$

where ${k}_{{{{{{\rm{d}}}}}}}$ is a dimensional coefficient of erodibility of the channel bed, *m* and *n* are dimensionless empirically derived constants of erosion exponents for the shear stress exerted on channel beds (which are generally positive with the $\frac{m}{n}$ ratio of ~0.5^104^), *PA* is a proxy for water discharge that numerically integrates the total area and precipitation from upstream connected nodes,∇*Z* is land surface slope, ${k}_{{hl}}$ is the diffusion coefficient with different values for terrestrial and marine environments, and *z* is elevation. Values of these model parameters are listed in Supplementary Table 4.

## Supplementary information

Supplementary Information

Description of Additional Supplementary Files

Supplementary Movie 1

Supplementary Movie 2

Supplementary Movie 3

Supplementary Movie 4

Supplementary Movie 5

Supplementary Movie 6

## Data Availability

The crustal structure, temperatures, and mantle traction field data can be accessed at https://pubs.geoscienceworld.org/gsa/geosphere/article/14/3/1207/530582/Reconstruction-modeling-of-crustal-thickness-and, https://www.nature.com/articles/s41467-019-12950-8#Sec14, and https://www.nature.com/articles/s41467-022-31903-2.

## Code availability

The original version of the codes used to perform the simulations in this study can be accessed at https://github.com/underworldcode/UWGeodynamics and https://aspect.geodynamics.org. The codes used to make the figures can be accessed at www.soest.hawaii.edu/gmt/ and www.paraview.org/.
