# Supplementary material for: The role of gravitational body forces in the development of metamorphic core complexes
Source: Nat Commun. 2022 Sep 26;13:5646. doi: 10.1038/s41467-022-33361-2 (PMC9513114; doi:10.1038/s41467-022-33361-2)
Supplement: Supplementary file 1 — Supplementary Information [file 41467_2022_33361_MOESM1_ESM.pdf]

Supplementary Information

for

The role of gravitational body forces in the development of metamorphic core complexes

Alireza Bahadori[1,2*], William E. Holt[2], Jacqueline Austermann[1], Lajhon Campbell[2], E. Troy Rasbury[2], Daniel M. Davis[2], Christopher M. Calvelage[3], Lucy M. Flesch[4]

[1]Lamont-Doherty Earth Observatory, Columbia University in the City of New York, Palisades, NY, USA. [2]Department of Geosciences, Stony Brook University, Stony Brook, NY, USA. [3]Department of Earth and Atmospheric Sciences, University of Houston, Houston, TX, USA. [4]Department of Earth, Atmospheric, and Planetary Sciences, Purdue University, West Lafayette, IN, USA. *Corresponding author: abahadori@ldeo.columbia.edu

Supplementary Tables

Supplementary Table 1. Model parameters applied in thermomechanical model.

Material	Density (kg/m^3)	Heat capacity ($J/^oK.kg$)	Heat diffusivity (m^2/s)	Radiogenic heat production (MW/m^3)	Flow law (dislocation creep)
Topography	2720	1000	1×10^{-6}	7.67×10^{-7}	Wet Quartz (Goetze[1])
Upper crust	2720	1000	1×10^{-6}	7.67×10^{-7}	Wet Quartz (Goetze[1])
Middle crust	2720	1000	1×10^{-6}	7.67×10^{-7}	Wet Quartz (Goetze[1])
Lower crust	2720	1000	1×10^{-6}	7.67×10^{-7}	Wet Quartz (Goetze[1])
Mantle lithosphere	3370	1000	1×10^{-6}	0.0	Dry Olivine (Brace and Kohlstedt[2])
Sediment	2300	1000	1×10^{-6}	0.0	Wet Quartz (Gleason and Tullis[3])
Air	1	1000	1×10^{-6}	0.0	5.0×10^{18} Pa s
Sticky air	1	1000	1×10^{-6}	0.0	5.0×10^{18} Pa s
Colorado Plateau - Middle-lower crust	2720	1000	1×10^{-6}	7.67×10^{-7}	Wet Quartz (Goetze[1])

Supplementary Table 2. Model parameters of rheology applied in thermomechanical model.

Flow law (dislocation creep)	A_0 (Mpa^n/S)	n	E_0 (J/mole)	V_0 (m^3/mole)	R ($J/mole^oK$)	f
Dry Olivine	7.0×10^4	3	5.2×10^5	0	8.3144	1.5
Wet Quartz	5.0×10^{-6}	3	1.9×10^5	0	8.3144	1.0, 20

Supplementary Table 3. Model parameters of partial melting processes applied in thermomechanical model.

Parameter	Value
Latent heat of fusion (kJ/kg)	250
Melt fraction density change (k)	0.13
Solidus coefficient 'a_s' (k)	993
Solidus coefficient 'b_s' (k/Pa)	$-1.2e^{-7}$
Solidus coefficient 'c_s' (k/Pa)	$1.2e^{-16}$
Liquidus coefficient 'a_l' (k)	1493
Liquidus coefficient 'b_l' (k/Pa)	$-1.2e^{-7}$
Liquidus coefficient 'c_l' (k/Pa^2)	$1.6e^{-16}$
Melt viscous softening factor	$1.0e^{-2}$
Viscous softening melt fraction	0.15-0.3

Supplementary Table 4. Model parameters applied in surface processes simulation.

**Definition**	**Value**	**Symbol (unit)**
Exponent in stream-power law	0.5	m
Exponent in stream-power law	1.0	n
Erosion coefficient of fluvial process	4×10^{-7}	K_d (yr^{-1})
Minimum precipitation	0.25	P_{min} ($m\ yr^{-1}$)
Maximum precipitation	1.25	P_{max} ($m\ yr^{-1}$)
Maximal elevation for computing linear precipitation trend	2700	Z (m)
Terrestrial diffusion coefficient for hillslope processes	0.5	K_{hl} ($m^2\ yr^{-1}$)
Marine diffusion coefficient for hillslope processes	1	K_{hl} ($m^2\ yr^{-1}$)

Supplementary Figures

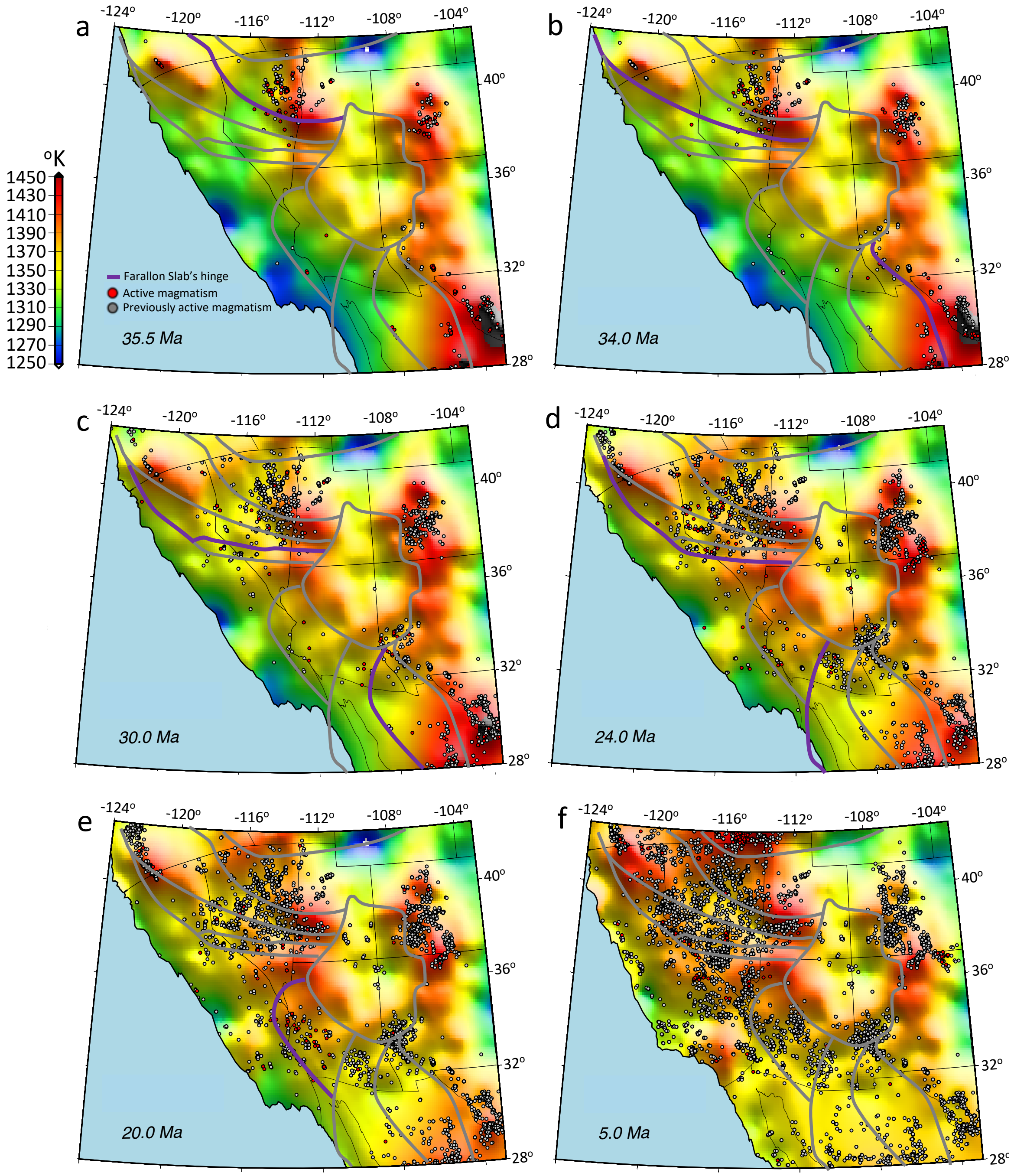

Supplementary Figure 1. Paleo-magmatism evolution associated with the Farallon Slab rollback in southwestern North America since the late Eocene. a, Timing and placement of Cenozoic magmatic patterns from Bahadori and Holt[4] and the inferred temperature changes at 100 km depth in southwestern North America at 35.5 Ma. The closed gray line represents the present-day boundary of the Colorado Plateau. Red dots represent active paleo-magmatism for the time shown in each panel, gray dots represent previously active paleo-magmatism, gray lines represent the location of the Farallon Slab's hinge during slab rollback from Bahadori and Holt[4], and purple lines represent the location of the Farallon Slab's hinge for the time shown in each panel; **b-f,** Similar to 'a' but at 34, 30, 24, 20, and 5 Ma, respectively. Palinspastic reconstruction of state boundaries is from Bahadori and Holt[4]. The map images were created by authors using: www.soest.hawaii.edu/gmt/.

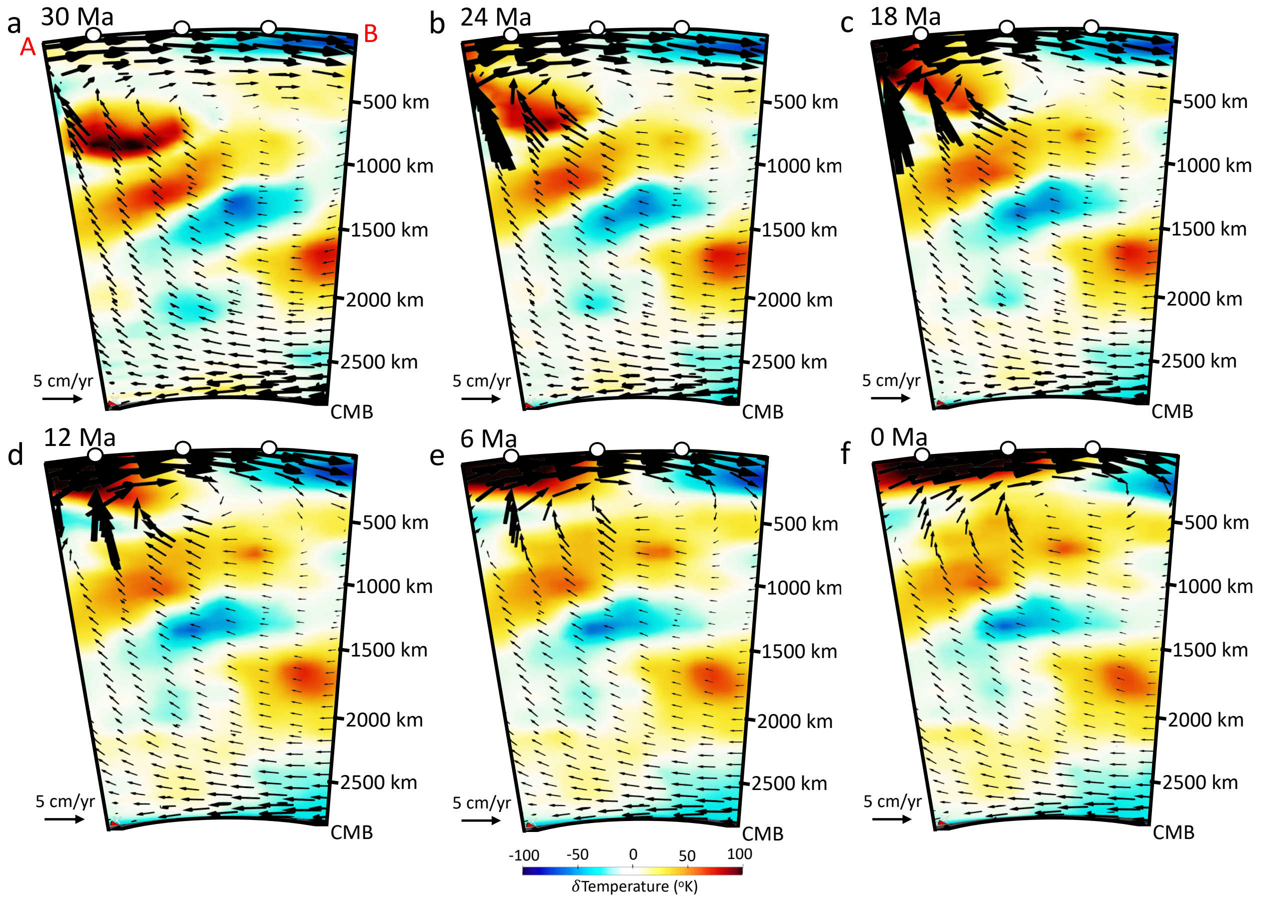

Supplementary Figure 2. Evolution of mantle thermal structures beneath the Basin and Range Province since the late Eocene. a-f, Radial cross-sections of the reconstructed mantle temperature variations from surface to core-mantle boundary (CMB) along the line A–B at latitude 38ºN in Supplementary Figure 3. Superimposed on these cross-sections are the corresponding mantle flow velocity vectors. The vectors are extracted from global flow field in the mantle's no-net rotation (NNR) frame of reference along a cross section fixed relative to North America. Panels show landward intrusion of hot anomalies within the Basin and Range Province.

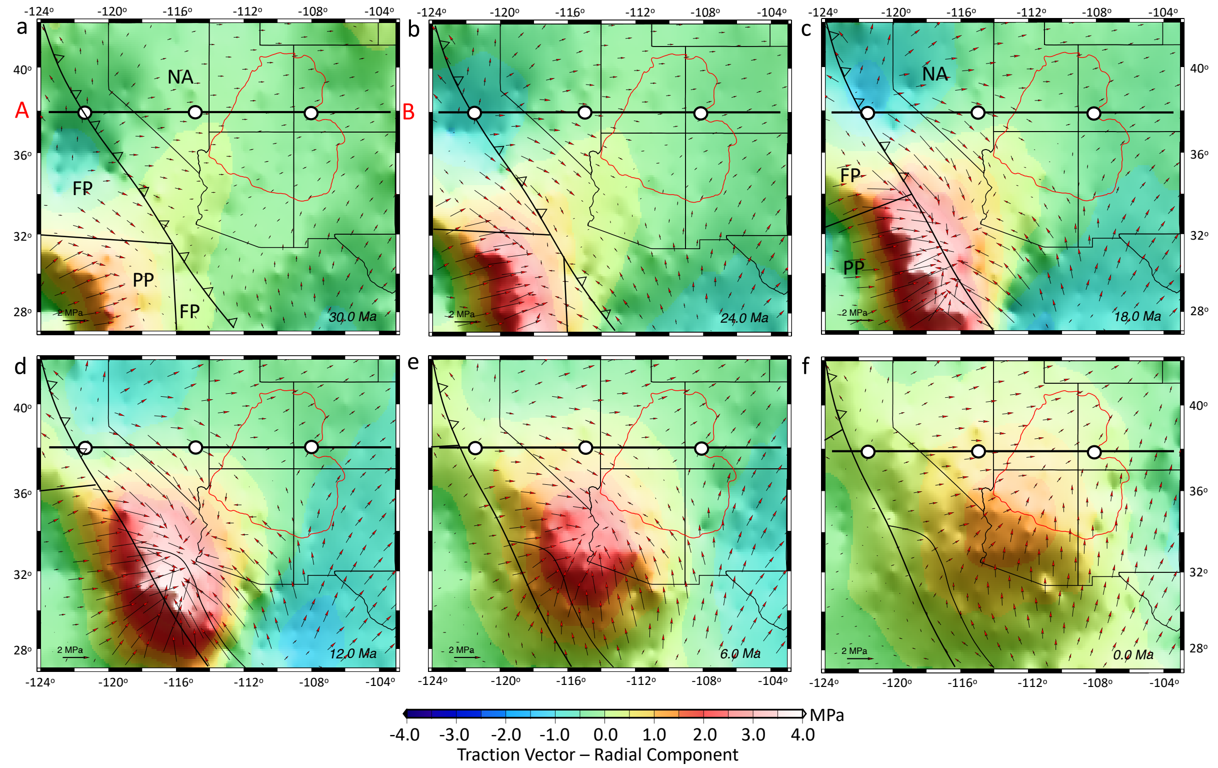

Supplementary Figure 3. Evolution of the traction field associated with mantle convection below the North American lithosphere since the late Eocene. a-f, Vectors represent horizontal components of the traction vector field below the lithosphere in southwestern North America, and contours represent the radial component of the traction vector field at 30, 24, 18, 12, 6, and 0 Ma, respectively. The closed red line indicates the present-day edge of the Colorado Plateau. FP: Farallon Plate; PP: Pacific Plate; NA: North American plate. The map images were created by authors using: www.soest.hawaii.edu/gmt/.

Supplementary References:

1. Goetze, C. The mechanisms of creep in olivine. *Philosophical Transactions of the Royal Society of London. Series A, Mathematical and Physical Sciences* **288**, 99-119 (1978).

2. Brace, W.F. & Kohlstedt, D.L. Limits on lithospheric stress imposed by laboratory experiments. *Journal of Geophysical Research: Solid Earth* **85**, 6248-6252 (1980).

3. Gleason, G.C. & Tullis, J. A flow law for dislocation creep of quartz aggregates determined with the molten salt cell. *Tectonophysics* **247**, 1-23 (1995).

4. Bahadori, A. & Holt, W. E. Geodynamic evolution of southwestern North America since the Late Eocene. *Nat. Commun.* **10**, 1–18 (2019).
