# Supplementary material for: The role of gravitational body forces in the development of metamorphic core complexes
Source: Nat Commun. 2022 Sep 26;13:5646. doi: 10.1038/s41467-022-33361-2 (PMC9513114; doi:10.1038/s41467-022-33361-2)
Supplement: Supplementary file 2 — Description of Additional Supplementary Files [file 41467_2022_33361_MOESM2_ESM.pdf]

## Description of Additional Supplementary Files

File name: Supplementary Movie 1

Description: Deformation of the model with a crustal root and a laterally varying surface elevation since the late Eocene under the influence of basal tractions, free slip side boundaries, and non-uniform distribution of gravitational body forces. Left: the evolution of crustal structure together with the magnitude of the finite plastic strain for the brittle upper crust through time; Right: the evolution of finite strain (values represent second invariant of strain tensor) magnitude through the brittle and ductile zones of the crust. Note that in the experiment with a crustal root the weakening and damage of the brittle upper crust and the resulting accumulated plastic strain and plastic shear zones produce basins and ranges. A necking center below the highlands forms and remains active. Dip of the active shear zones is high at the onset of topographic collapse and evolves into low-angle detachment fault through time.

File name: Supplementary Movie 2

Description: Deformation of the model with a flat Moho topography and a laterally varying surface elevation since the late Eocene under the influence of basal tractions, free slip side boundaries, and non-uniform distribution of gravitational body forces. Left: the evolution of crustal structure together with the magnitude of the plastic strain/failure for the brittle upper crust through time; Right: the evolution of finite strain (values represent second invariant of strain tensor) magnitude at the brittle and ductile zones of the crust. Note that in the experiment with a flat Moho topography and a laterally varying surface elevation the weakening and damage of the brittle upper crust and the resulting accumulated plastic strain and plastic shear zones produce basins and ranges. Necking centers in the crust remain active, and the conjugate shear zones are diffused. Dips of the active shear zones remain high and no low-angle detachment fault or metamorphic core complex forms.

File name: Supplementary Movie 3

Description: Deformation of the model with a uniform thickness crust (flat Moho topography and flat surface elevation) since the late Eocene under the influence of basal tractions, free slip side boundaries, and uniform distribution of gravitational body forces. Left: the evolution of crustal structure together with the magnitude of the plastic strain/failure for the brittle upper crust through time; Right: the evolution of finite strain (values represent second invariant of strain tensor) magnitude at the brittle and ductile zones of the crust. Note that in the experiment with a uniform thickness crust there is no weakening and damage of the brittle upper crust in response to basal tractions at the base, free slip side boundary conditions, and uniform distribution of gravitational body forces. Hence, the crustal structure experiences minimal defamation for the whole duration of simulation.

File name: Supplementary Movie 4

Description: Deformation of the model with a crustal root and a laterally varying surface elevation since 30 Ma under the influence of left side velocity boundary condition, equal pressure at the base, and non-uniform distribution of gravitational body forces. Left: the evolution of crustal structure

together with the magnitude of the finite plastic strain for the brittle upper crust through time; Right: the evolution of finite strain (values represent second invariant of strain tensor) magnitude through the brittle and ductile zones of the crust. Strain develops in response to extension and fault rotation. Note that in the experiment with a crustal root, a reduction in crustal thickness in response to isostatic rebound of the crustal root and collapse of topography increases the local strains within a necking zone which accommodates the doming and exhumation of the middle crust at the surface.

File name: Supplementary Movie 5

Description: Deformation of the model with a flat Moho topography and a laterally varying surface elevation since 30 Ma under the influence of left side velocity boundary condition, equal pressure at the base, and non-uniform distribution of gravitational body forces. Left: the evolution of crustal structure together with the magnitude of the plastic strain/failure for the brittle upper crust through time; Right: the evolution of finite strain (values represent second invariant of strain tensor) magnitude at the brittle and ductile zones of the crust. Note that in the experiment with a flat Moho topography and a laterally varying surface elevation the weakening and damage of the brittle upper crust and the resulting accumulated plastic strain and plastic shear zones produce basins and ranges and symmetric domes in the crust. The right limb of active shear zones remain at high-angle dips and the left limb evolves into a low-angle detachment fault, but without metamorphic core complex formation.

File name: Supplementary Movie 6

Description: Deformation of the model with a uniform thickness crust (flat Moho topography and flat surface elevation) since 30 Ma under the influence of left side velocity boundary condition, equal pressure at the base, and uniform distribution of gravitational body forces. Left: the evolution of crustal structure together with the magnitude of the plastic strain/failure for the brittle upper crust through time; Right: the evolution of finite strain (values represent second invariant of strain tensor) magnitude at the brittle and ductile zones of the crust. Note that in the experiment with a uniform thickness crust and flat Moho topography the weakening and damage of the brittle upper crust and the resulting accumulated plastic strain and plastic shear zones produce basins and ranges, but no doming or metamorphic core complex formation. Necking centers in the crust remain active, and the conjugate shear zones are diffuse or distributed throughout. Dips of the active shear zones remain high and no low-angle detachment fault forms.
